# Metabolic Profiling at COVID-19 Onset Shows Disease Severity and Sex-Specific Dysregulation

**DOI:** 10.3389/fimmu.2022.925558

**Published:** 2022-06-30

**Authors:** Francisco C. Ceballos, Ana Virseda-Berdices, Salvador Resino, Pablo Ryan, Oscar Martínez-González, Felipe Peréz-García, María Martin-Vicente, Oscar Brochado-Kith, Rafael Blancas, Sofía Bartolome-Sánchez, Erick Joan Vidal-Alcántara, Oihane Elena Albóniga-Díez, Juan Cuadros-González, Natalia Blanca-López, Isidoro Martínez, Ignacio Ramirez Martinez-Acitores, Coral Barbas, Amanda Fernández-Rodríguez, María Ángeles Jiménez-Sousa

**Affiliations:** ^1^ Unit of Viral Infection and Immunity, National Center for Microbiology (CNM), Health Institute Carlos III (ISCIII), Madrid, Spain; ^2^ Centro de Investigación Biomédica en Red de Enfermedades Infecciosas (CIBERINFEC), Instituto de Salud Carlos III, Madrid, Spain; ^3^ Department of Infectious Diseases, Hospital Universitario Infanta Leonor, Madrid, Spain; ^4^ Critical Care Department, Hospital Universitario del Tajo, Aranjuez, Spain; ^5^ Universidad Alfonso X el Sabio, Villanueva de la Cañada, Madrid, Spain; ^6^ Clinical Microbiology Department, Hospital Universitario Príncipe de Asturias, Alcalá de Henares, Spain; ^7^ Department of Biomedicine and Biotecnology, Faculty of Medicine, University of Alcalá de Henares, Alcalá de Henares, Spain; ^8^ Centre for Metabolomics and Bioanalysis (CEMBIO), Department of Chemistry and Biochemistry, Facultad de Farmacia, Universidad San Pablo-CEU, CEU Universities, Urbanización Montepríncipe, Madrid, Spain; ^9^ Allergology Department, University Hospital Infanta Leonor, Madrid, Spain

**Keywords:** COVID-19, metabolomics, severity, sex-specific, Spanish hospitals, disease onset

## Abstract

**Background:**

metabolic changes through SARS-CoV-2 infection has been reported but not fully comprehended. This metabolic dysregulation affects multiple organs during COVID-19 and its early detection can be used as a prognosis marker of severity. Therefore, we aimed to characterize metabolic and cytokine profile at COVID-19 onset and its relationship with disease severity to identify metabolic profiles predicting disease progression.

**Material and Methods:**

we performed a retrospective cross-sectional study in 123 COVID-19 patients which were stratified as asymptomatic/mild, moderate and severe according to the highest COVID-19 severity status, and a group of healthy controls. We performed an untargeted plasma metabolic profiling (gas chromatography and capillary electrophoresis-mass spectrometry (GC and CE-MS)) and cytokine evaluation.

**Results:**

After data filtering and identification we observed 105 metabolites dysregulated (66 GC-MS and 40 CE-MS) which shown different expression patterns for each COVID-19 severity status. These metabolites belonged to different metabolic pathways including amino acid, energy, and nitrogen metabolism among others. Severity-specific metabolic dysregulation was observed, as an increased transformation of L-tryptophan into L-kynurenine. Thus, metabolic profiling at hospital admission differentiate between severe and moderate patients in the later phase of worse evolution. Several plasma pro-inflammatory biomarkers showed significant correlation with deregulated metabolites, specially with L-kynurenine and L-tryptophan. Finally, we describe a strong sex-related dysregulation of metabolites, cytokines and chemokines between severe and moderate patients. In conclusion, metabolic profiling of COVID-19 patients at disease onset is a powerful tool to unravel the SARS-CoV-2 molecular pathogenesis.

**Conclusions:**

This technique makes it possible to identify metabolic phenoconversion that predicts disease progression and explains the pronounced pathogenesis differences between sexes.

## Background

Severe acute respiratory syndrome coronavirus 2 (SARS-CoV-2) causes an acute respiratory disease, the coronavirus disease 2019 (COVID-19), which has been associated with high mortality and morbidity rates worldwide (1). COVID-19 presents a wide range of symptoms (2). Around 80% of COVID-19 patients develop mild-to-moderate, 15% severe, and 5% critical illness (3). A cytokine storm (deregulated pro-inflammatory response) usually appears in severe COVID-19 patients, with frequent episodes of thromboembolism, which are strongly associated with acute respiratory distress syndrome (ARDS), sepsis, and mortality (3). However, the biological mechanisms of SARS-CoV-2 induced pathology are still not completely understood.

Although recent studies have shown that SARS-CoV-2 vaccines are about 80% effective at preventing hospital admission of COVID-19 patients (4), there is still an urgent need for drug treatment due to the unequal vaccination rates around the world (5). Understanding the relationship between biochemical changes and COVID-19 severity is a crucial first step towards new therapeutic strategies.

Several studies have revealed metabolic dysregulation during COVID-19 progression (6–17). These metabolic changes affect multiple organs during SARS-CoV-2 infection (15), which can be used as a prognostic marker. However, there is significant heterogenicity among studies published in experimental design, sample size, the origin of the patients, timing of sampling, and technology used. Unlike other omic approaches, metabolomic results are not usually comparable across different studies due to differences in separation techniques and data acquisition (6). For this reason, the use of complementary techniques with an untargeted approach could shed more light on the biological and biochemical alterations produced by SARS-CoV-2 for future research.

Additionally, it is essential to use different separation techniques, like gas chromatography-mass spectrometry (GC-MS), which has been widely employed, or capillary electrophoresis-mass spectrometry (CE-MS), which has been much less previously addressed in COVID-19, to obtain a broader range of metabolites to better understand the dysregulation associated to SARS-CoV-2. Moreover, analysis of alternative populations from early-onset to varying degrees of COVID-19 severity and new approaches are needed, such as the relationship between sex and metabolism in the context of SARS-CoV-2 infection. Therefore, additional studies analyzing the metabolic profile of the COVID-19 disease are needed to keep delving into the disease etiology and discover new targets for drug development. Besides, metabolic processes can influence the function of immune cells (18), being key to the emergence of immunometabolism. Leukocytes can generate and to be influenced by key metabolites important to innate and adaptive immunity. An example of this is the tryptophan-kynurenine pathway, which shows a modulatory effect on the immune responses. *In vitro* experiments have shown that tryptophan deprivation inhibits proliferation of T cells, sensitizes T cells to apoptosis, and plays a role in CD8 T-cell suppression, being the tryptophan metabolism an essential regulator of inflammation and immunity (19). Thus, integration of metabolomics with other systems biology platforms to study immunological responses will enhance the knowledge of molecular mechanisms implicated in COVID-19 (20).

Therefore, this study aimed to characterize the metabolic and cytokine profile of COVID-19 patients at hospital admission and identify its association with the highest COVID-19 severity reached, as well as the differences by sex.

## Methods

### Study Subjects

We performed a retrospective study in 123 COVID-19 patients enrolled from March to September 2020 at three hospitals in Madrid: Infanta Leonor University Hospital, del Tajo University Hospital, and Príncipe de Asturias University Hospital. The study protocol was approved by the Ethics Committee of the Institute of Health Carlos III (PI 33_2020-v3) and the Ethics Committee of each hospital.

Patients were classified according to their highest COVID-19 severity status (**Supplementary Data 1**): 1) asymptomatic/mild who had minor or no COVID-19 symptoms. 2) moderate who required hospitalization but did not fulfill severe COVID-19 criteria. 3) severe who had any of the following criteria: i) death during hospitalization, ii) ICU admission, iii) invasive mechanical ventilation, or iv) presence of bilateral pulmonary infiltrates, non-invasive mechanical ventilation, and oxygen saturation (Sat02) <93%. Besides, a control group of 15 pre-pandemic healthy controls without any known infection was included. The STROBE-ID checklist was used to strengthen the design and conduct the study.

### Clinical Data and Sample Collection

Epidemiological and clinical data were prospectively collected from clinical records using an electronic case report form (eCRF) built using REDCap.

Patient samples were collected at hospital admission or within the first days after hospitalization (median = 2 days) and before treatment with immunotherapy against IL-6 (e.g., Tocilizumab), interferon beta, corticoids, or ribavirin, among others. Plasma samples were obtained after centrifugation blood in EDTA tubes and stored at -80°C.

### Outcome Variables

The primary outcomes were SARS-CoV-2 infection (COVID-19 patients versus healthy controls), COVID-19 symptomatology (hospitalized symptomatic patients (moderate plus severe) versus asymptomatic), and COVID-19 severe (severe versus moderate).

### Sample Treatment for Non-Targeted Metabolomics

Plasma samples were inactivated with cold (-20°C) MeOH: EtOH (1:1, v/v) and vortex-mixed for 1 min, incubated on ice for 5 min, and centrifuged for 20 min at 16000 *xg* at 4°C. The resulting supernatant was stored at -80°C until analysis. Due to the broad differences in physical-chemical properties of metabolites, we used GC-MS (focused on small molecules that can be made volatile by derivatization) and CE-MS (focused on polar and ionic compounds) in order to increase the metabolite coverage. Sample preparation for GC-MS and CE-MS was carried out at the Centre for Metabolomics and Bioanalysis (CEMBIO, Madrid, Spain), based on previously developed methods (21, 22). Quality controls (QC) samples were regularly analyzed through the run to assess the data quality for each platform by pooling and mixing equal volumes of each corresponding sample. Also, a pair of blank solutions were analyzed at the beginning and the end of each analytical sequence (full description in **Supplementary Data 2**).

### Data Treatment and Quality Assurance

For GC-MS, the alignment was carried out with Agilent Mass Profiler Professional version 15.1 and exported into Agilent MassHunter Quantitative Analysis version 10.0 to assign target ions and obtain the compound abundances.

For CE-MS, raw data were processed with MassHunter Profiler software (version 10.0) applying the molecular feature extraction (MFE). All features extracted by MFE were aligned across all samples with the batch recursive feature extraction. The continuously infused references masses (*m/z* 121.0509 and *m/z* 922.0098) and those features found in blanks were excluded from the final list.

For both GC-MS and CE-MS analysis, metabolites with poor reproducibility (coefficient of variation (CV) in the QCs greater than 30%) and those features not presented in 70% of samples in at least one sample group were discarded. Regarding GC-MS, the final concentration of each metabolite was also normalized according to the IS abundance. Finally, the QC intensity drop was corrected, and the matrixes were further used for statistical analysis (full description in **Supplementary Data 2**).

### Determination of Inflammatory Markers

Twenty-six biomarkers previously related to COVID-19 (**Supplementary Data 3**) were measured with a custom ProcartaPlex multiplex immunoassay (Invitrogen) following the manufacturer’s specifications using the Luminex 200TM system (BioRad Laboratories Hercules, California, USA). We used the raw fluorescence intensity (FI) values as a relative quantification of the analyte abundances, as previously described (23).

### Statistical Analysis

For descriptive data, differences between groups were tested using Chi-square or Fisher’s exact test with Monte Carlo simulated p-value for categorical data and Kruskal-Wallis test for continuous variables. Before the statistical analysis, metabolomic data were base 2 log-transformed (log2) and auto-scaled. Regarding plasma markers, FI values were pre-processed with a weighted Box-Cox, followed by quantile normalization (24).

Exploratory analysis was performed using a volcano plot approximation, where the logarithm of the fold change was compared to the *p*-value of a non-parametric Kruskal Wallis test, and metabolite concentration heatmaps (for GC-MS and CE-MS independently). A paired supervised multivariate model by partial least squares - discriminant analysis (PLS-DA) was also used separately for GC-MS and CE-MS data matrices. Cross-validation with leave-one-out cross-validation (LOOCV) and R^2^ and Q^2^ values as performance measures were used. Variable importance in projection (VIP) score for each feature was obtained from the PLS-DA model. The validity of the model was confirmed by a permutation out by separation distance (Between-group sum of squares and Within-group sum of squares (B/W)) with a permutation number of 1000. Prediction areas in pairwise severity-group classification were obtained using the Mahalanobis distance (25). Additionally, multivariable logistic regression models were used to study the association of the plasma metabolites with the three primary outcomes. Age and sex were used as covariables. In all cases, *p*-values were corrected for multiple testing using the false discovery rate (FDR) with Benjamini and Hochberg procedure. Those metabolites with VIP ≥1 from PLS-DA or *q*-value <0.1 from regression models were considered significant. Pearson correlation was used to investigate the relationship between significant metabolites and plasma markers of inflammation. To make correlations more visual, we employed the chord diagram technique that is useful in visualizing inter-relationships between large numbers of variables. In this study, the variables were the markers of interest (metabolites and cytokines) and the relationship between them was the Pearson’s correlation coefficient.

The interaction between metabolites and plasma biomarkers with sex was tested using a linear model by testing both variables and their interaction. Independent models for each sex were used for those metabolites that exhibited significant interaction with the sex variable. Confounding effects between severity, biological sex, demographic covariables and comorbidities were assessed using the Cochran-Mantel-Haenszel test.

Analyses were performed using MetaboAnalyst 4.0 software and the R 4.0.3 software. Two-sided tests were used for all statistical methods.

## Results

### Characteristics of Participants

Clinical, epidemiological, and biochemical data of 15 healthy controls and 123 COVID-19 patients (12 asymptomatic/mild, 64 moderate, and 47 severe) are summarized in **Table 1**. No statistically significant association was found between covariates and disease severity. We identified differences for some interventions that were performed after sample collection, such as tocilizumab (Fisher’s exact test = 8.1e-05), corticosteroids (Fisher’s exact test = 5.7e-08), and the use of supplemental oxygen (Fisher’s exact test = 3.2.e-07). Only the administration of cloroquine/hydroxycloroquine (Fisher’s exact test = 1.6e-05) was performed before sample collection for 18 patients, where no differences were found with respect to moderate and severe groups (p=0.980).

**Table 1:** Patient’s characteristics. Patients were classified according to the highest disease severity reached during the COVID-19 evolution.

	COVID-19 Severity			
	Healthy	Asymptomatic/Mild	Moderate	Severe
**Demographics**				
N	15	12	64	47
Age	63.9 ± 8.3	59.7 ± 18.2	61.1 ± 15.3	63.3 ± 18.1
Gender (male)	7/15 (46.7%)	4/12 (33.3%)	34/64 (53.1%)	33/47 (70.2%)
BMI>=25	8/11 (72.7%)	2/12 (16.6%)	11/64 (17.1%)	13/47 (27.6%)
Smoke status (Yes)	NA	2/12 (16.7%)	4/64 (4.4%)	3/47 (6.3%)
Former smoker	NA	4/12 (33.3%)	8/64 (11.7%)	11/47 (23.4%)
**Comorbilities**				
Hypertension	NA	5/12 (41.6%)	27/64 (42.2%)	23/47 (48.9%)
Cardiopathy	NA	4/12 (30.7%)	12/64 (17.6)	8/47 (17.0%)
Chronic pulmonary disease	NA	0/12 (0%)	4/64 (6.3%)	10/47 (21.2%)
Chronic kidney disease	NA	2/12 (16.7%)	4/64 (6.3%)	9/47 (19.1%)
Chronic liver disease	NA	NA	3/64 (4.6%)	2/47 (4.2%)
Chronic neurological disease	NA	0/12 (0%)	9/64 (14.1%)	8/47 (17.0%)
Neoplasia	NA	NA	3/63 (4.8%)	4/47 (8.5%)
Diabetes	NA	2/12 (15.7%)	10/64 (23.4%)	11/47 (23.4%)
Chronic inflammatory disease	NA	NA	2/63 (3.1%)	5/47 (10.6%)
Autoimmune disease	NA	NA	1/63 (1.5%)	4/47 (8.5%)
**Therapy**				
**Chronic medications**				
NSAIDs	NA	0/12 (0%)	0/64 (0%)	7/47 (14.9%)
ACE inhibitors	NA	0/12 (0%)	15/64 (22.33.4%)	11/47 (23.4%)
ARBs	NA	1/12 (8.3%)	7/64 (10.9%)	8/47 (17.0%)
Corticosteroids	NA	0/12 (0%)	6/64 (9.4%)	6/47 (12.8%)
HIV antiretrovirals	NA	0/12 (0%)	1/64 (1.6%)	1/47 (2.1%)
**Treatment**				
Chloroquine and hidroxychloroquine	NA	NA	62/63 (98.4%)	33/47 (70.2%)
Tocilizumab	NA	NA	10/64 (15.6%)	20/47 (42.5%)
Corticosteroids	NA	NA	26/64 (40.6%)	40/47 (85.1%)
**COVID-19 related symptoms**				
Dyspnoea	0/15 (0%)	0/12 (0%)	41/64 (64.0%)	41/47 (87.2%)
Cough	0/15 (0%)	0/12 (0%)	48/63 (76.1%)	34/47 (72.3%)
Headache	0/15 (0%)	0/12 (0%)	24/63 (38.1%)	10/47 (21.2%)
Diarrhea or abdominal pain	0/15 (0%)	0/12 (0%)	33/63 (52.4%)	19/47 (40.4%)
**Hospitalization**				
Length of stay (days)	0/15 (0%)	0/12 (0%)	10.9 ± 8.7	22.1 ± 15.6
Maximum Temperature	NA	NA	37.5 ± 0.8	38.2 ± 0.8
Oxygen therapy	0/15 (0%)	0/12 (0%)	37/64 (57.3.8%)	47/47 (100%)
Non-Invasive mechanical ventilation	0/15 (0%)	0/12 (0%)	3/64 (4.6%)	20/47 (38.99%)
Invasive mechanical ventilation	0/15 (0%)	0/12 (0%)	0/64 (0%)	18/47 (38.2%)
Pulmonary infiltrates	0/15 (0%)	0/12 (0%)	58/64 (90.6%)	47/47 (100%)
**ICU**				
ICU length of stay	0/15 (0%)	0/12 (0%)	0/64 (0%)	6.61 ± 13.5
Death	NA	0/12 (0%)	0/64 (0%)	18/47 (38.3%)

Numerator indicates the number of patients with available data. Individual characteristics were summarized using standard descriptive statistics: mean ± standard deviation for continuous variables and count (percentage) for categorical variables. Differences between groups were tested using Fisher’s exact test. No statistically significant association was found between covariates and disease severity with the exception of the following treatments: Chloroquine/hydroxychloroquine (Fisher’s exact test = 1.6e^-05^), Tocilizumab (Fisher’s exact test = 8.1e^-05^), corticosteroids (Fisher’s exact test = 5.7e^-08^), and the use of supplemental oxygen (Fisher’s exact test = 3.2.e^-07^). NA, non-available; BMI, body mass index; NSAIDs, Nonsteroidal anti-inflammatory drugs; ACE, Angiotensin-converting-enzyme; ARBs, angiotensin II inhibitors; HIV, human immunodeficiency virus; ICU, intensive care unit.

No statistically significant effects were found between sexes, severity and other confounding variables (**Supplementary Data 4**).

### Metabolic Profiling of Plasma Samples

A total of 202 features were detected (66 GC-MS and 136 CE-MS). After data pre-processing and metabolite identification, 105 metabolites were used in the subsequent analysis (65 GC-MS and 40 CE-MS). Raw data is available upon request to corresponding authors.

Heatmaps showed a different pattern of metabolites for each COVID-19 severity status in GC-MS (**Supplementary Data 5**) and CE-MS (**Supplementary Data 6**). An initial exploratory analysis showed differential metabolites expression levels for each comparison, where COVID-19 patients and healthy controls were the most different (**Supplementary Data 7**).

PLS-DA showed that samples clustered in the different groups of patients with four principal components for GC-MS (R^2^ = 0.753 and Q^2^ = 0.623) and three principal components for CE-MS (R^2^ = 0.755 and Q^2 =^ 0.638) (**Supplementary Data 8**). In both GC-MS and CE-MS, asymptomatic/mild and healthy controls were perfectly separated from each other, as well as from moderate and severe patients. This indicates that three different metabolic profiles were perfectly distinguished in healthy controls, asymptomatic/mild, and severe/moderate patients. Even though moderate and severe patients were not perfectly clustered and separated, a clear tendency was observed, meaning several similarities in their metabolic profile.

**Figure 1:**
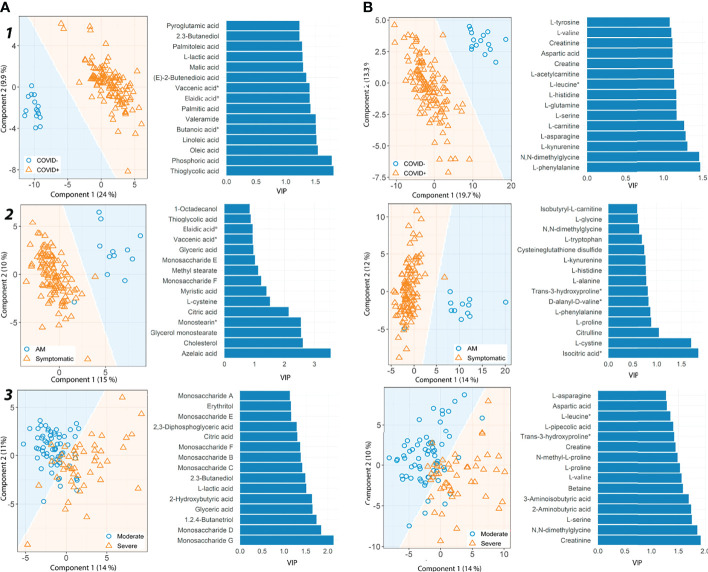
Partial least square discriminant analysis (PLS-DA) results for metabolites detected by GC-MS and CE-MS. **(A)** GC-MS metabolites, **(B)** CE-MS metabolites. For each group pairwise comparison, two different results are shown, prediction area visualization through the Mahalanobis distance and the first fifteen variable importance projections (VIP) scores. Each analysis is shown for different pairs of patient groups. ***1***) COVID+ patients vs. healthy controls. ***2***) Asymptomatic/Mild patients (AM) vs. Symptomatic patients and ***3***) Severe vs. Moderate patients. AM, Asymptomatic/Mild patients. Vaccenic acid, (E)-11-Octadecenoic acid. Elaidic acid, (E)-Oleic acid. Butanoic acid*, Butanoic acid. 2-(methoxyimino)-3-methyl-. trimethylsilyl ester. Monostearin*, Monostearin/1-stearoyl-rac-glycerol. L-leucine*, L-Leucine/Isoleucine. Trans-3-hydroxyproline*, trans-3-hydroxyproline/trans-4-hydroxyproline/cis-4-Hydroxy-D-proline.

Multivariate analysis for each platform and comparison established a good pairwise classification of the different patient groups (**Figure 1**; **Supplementary Data 9**). Fifteen metabolites had high VIP scores in different patient pairwise comparison: vaccenic acid ((E)-11-Octadecenoic acid), elaidic acid ((E)-oleic acid) and N,N-dimethylglycine (COVID+ vs. COVID- and Symptomatic vs. asymptomatic/mild); 2,3-butanediol, L-lactic acid, aspartic acid, L-glutamine and L-tryptophan (COVID+ vs. COVID- and severe vs. moderate); 2-hydroxyisovaleric acid (2-hydroxy-3-methylbutyric acid/pentanoic acid 2-[(trimethylsilyl)oxy]-, trimethylsilyl ester), citric acid, glyceric acid, citrulline, isocitric acid (citric Acid/isocitric acid), cysteineglutathione disulfide, L-glycine and L-tryptophan (Symptomatic vs. asymptomatic/mild, and Severe vs. Moderate). Also, we found metabolites with high VIP scores throughout all the pairwise comparisons, as D-glucarate, L-histidine, L-kynurenine, and L-phenylalanine (**Figure 1**).

### Metabolome Dysregulation

#### COVID-19 Patients vs. Healthy Controls

**Figure 2:**
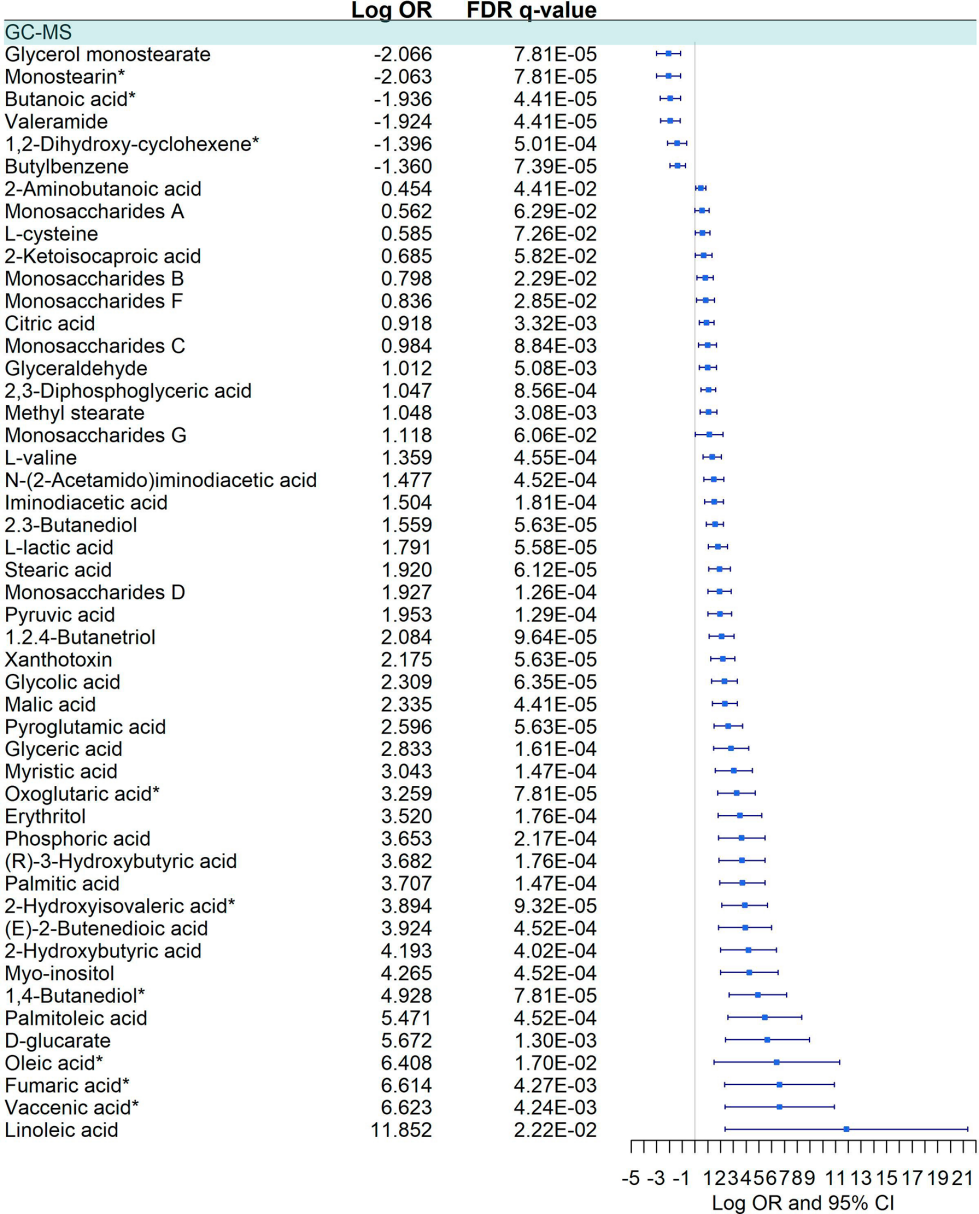
Pairwise comparison of GC-MS metabolites between COVID+ and COVID- individuals. Statistics: Logistic regression adjusted for age and sex was used. The False Discovery Rate (FDR) was used to cope with multiple testing, and q-values are provided. Monostearin*: Monostearin/1-stearoyl-rac-glycerol. Butanoic acid*: Butanoic acid. 2-(methoxyimino)-3-methyl-. trimethylsilyl ester. 1,2-Dihydroxy-cyclohexene*: 1.2-Bis(trimethylsiloxy)cyclohexene. Oxoglutaric acid*: Alpha-ketoglutaric acid. 2-Hydroxyisovaleric acid*: 2-Hydroxy-3-methylbutyric acid/Pentanoic acid 2-[(trimethylsilyl)oxy]-, trimethylsilyl ester. 1,4 - Butanediol*: (R*,S*)- 3,8-Dioxa-2,9-disiladecane, 2,2,9,9-tetramethyl-5,6-bis[(trimethylsilyl)oxy]. Oleic acid*: (Z)-Oleic Acid. Elaidic acid*: (E)-Oleic acid. Vaccenic acid*: (E)-11-Octadecenoic acid.

**Figure 3:**
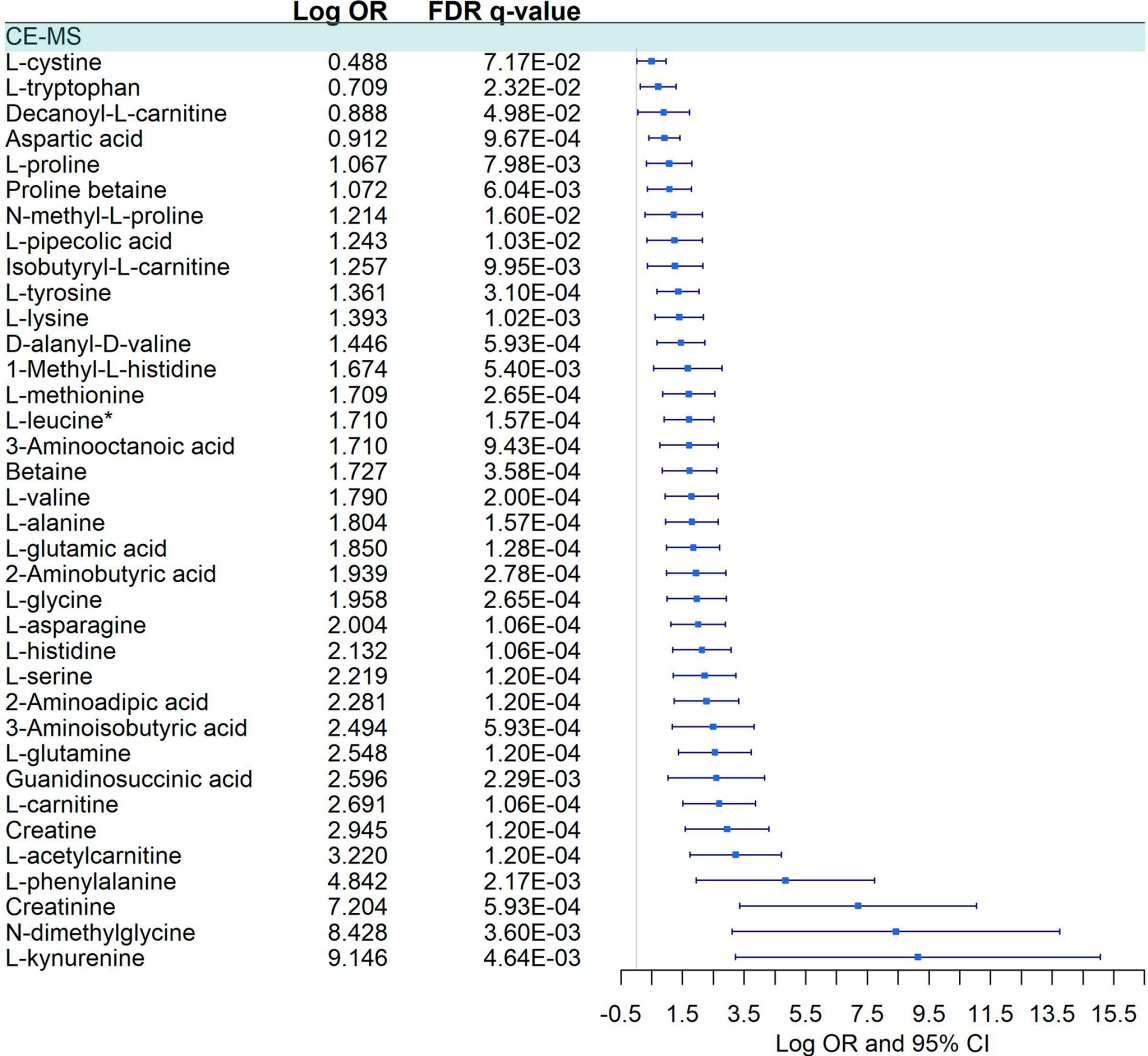
Pairwise comparison of CE-MS metabolites between COVID-19+ patients and COVID -. Statistics: Logistic regression adjusted for age and sex was used. The False Discovery Rate or FDR was used to cope with multiple testing, and q-values are provided. L-leucine*: L-Leucine/Isoleucine.

**Figure 4:**
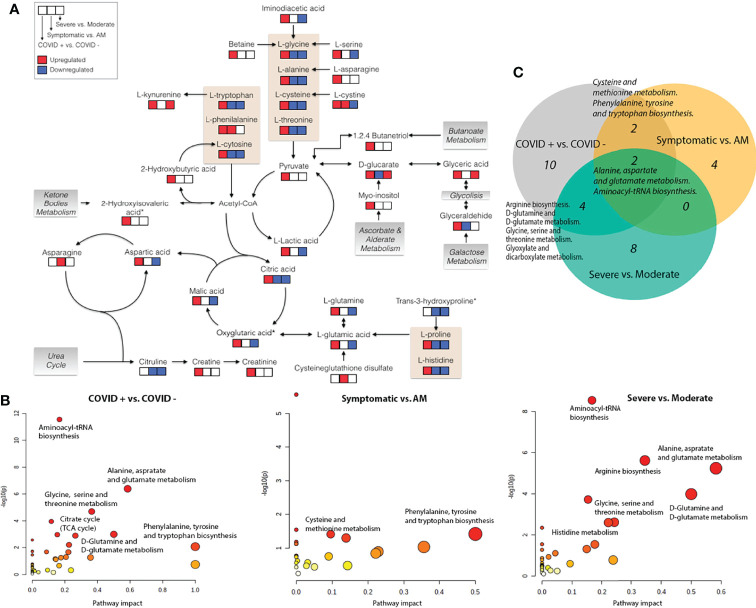
Metabolic pathways. **(A)** Schematic overview of key distributed metabolites and their metabolic relationships. Significant up (red) and down (blue) regulation are shown for the different severity status pairwise comparisons; **(B)** Pathway analysis based on enrichment analysis procedure. The most relevant metabolic pathways are represented according to their impact and adjusted *p*-value (obtained using MetaboAnalyst software v 4.0); **(C)** Venn diagram showing common significant metabolic pathways between different pairwise severity status comparisons. Oxoglutaric acid*, Alpha-ketoglutaric acid. 2-Hydroxyisovaleric acid*, 2-Hydroxy-3-methylbutyric acid/Pentanoic acid 2-[(trimethylsilyl)oxy]-, trimethylsilyl ester. Trans-3-hydroxyproline*, trans-3-hydroxyproline/trans-4-hydroxyproline/cis-4-Hydroxy-D-proline.

We detected a significant dysregulation between COVID-19 patients and healthy controls by multivariate logistic regression in 85 metabolites, 49 in GC-MS (**Figure 2**), and 36 in CE-MS (**Figure 3**). While six metabolites showed a significant reduction, most metabolites were significantly upregulated in COVID-19 patients compared to healthy controls (**Figures 2**, **3**). Metabolites with the higher VIP in the PLS-DA analysis (for both GC-MS and CE-MS approaches) were also significantly dysregulated (**Figures 2**, **3**), among which were amino acids, fatty acids, simple sugars, and organic acids. Enriched pathway analysis highlighted ten different metabolic pathways (**Figure 4**; **Supplementary Data 10**), involving mainly amino acid metabolism (L-phenylalanine, L-tyrosine, L-tryptophan, L-alanine, L-glutamine, L-serine, L-glycine, L-cysteine, L-arginine, L-valine, and L-leucine), along with energy and oxygen consumption metabolism (citrate cycle, pyruvate, and glycolysis pathways).

#### Symptomatic vs. Asymptomatic/Mild Patients

**Figure 5:**
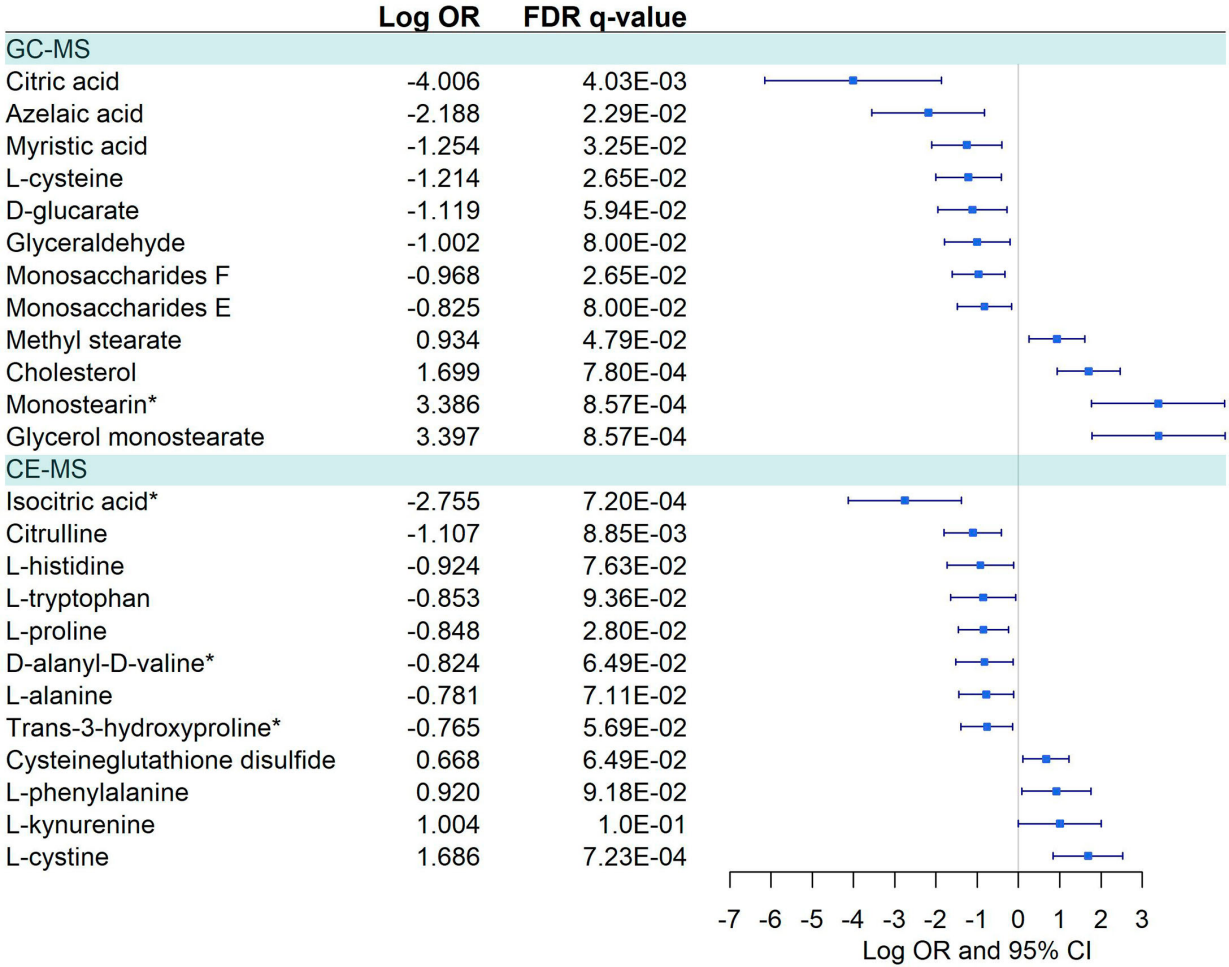
Pairwise comparisons between Symptomatic vs. Asymptomatic/Mild in GC-MS and CE-MS metabolites. Multivariate logistic regression, adjusted for age and sex, was used to compare Symptomatic vs. Asymptomatic/Mild. The False Discovery Rate or FDR was used to cope with multiple testing, q-values are provided. Monostearin*, Monostearin/1-stearoyl-rac-glycerol. Isocitric Acid*, Citric Acid/Isocitric acid. D-Alanyl-D-Valine*, D-Alanyl-D-Valine/Glycyl-L-leucine/N2-Acetyl-L-Lysine.

Twenty-three metabolites were dysregulated, 12 in GC-MS and 11 in CE-MS (**Figure 5**), mainly amino acids, fatty acids, and monosaccharides. Most of them exhibited downregulation in symptomatic patients, but some amino acids and various lipid-based metabolites such as monostearin, methyl stearate, cholesterol, and glycerol monostearate were upregulated. Four metabolic pathways were enriched using the significant 23 metabolites: three amino acid pathways and the aminoacyl-tRNA biosynthesis (ARS) pathway (**Figure 4**; **Supplementary Data 10**). These four pathways were also found among the dysregulated pathways in COVID-19 vs. healthy controls (**Figure 4**).

#### Severe vs. Moderate

**Figure 6:**
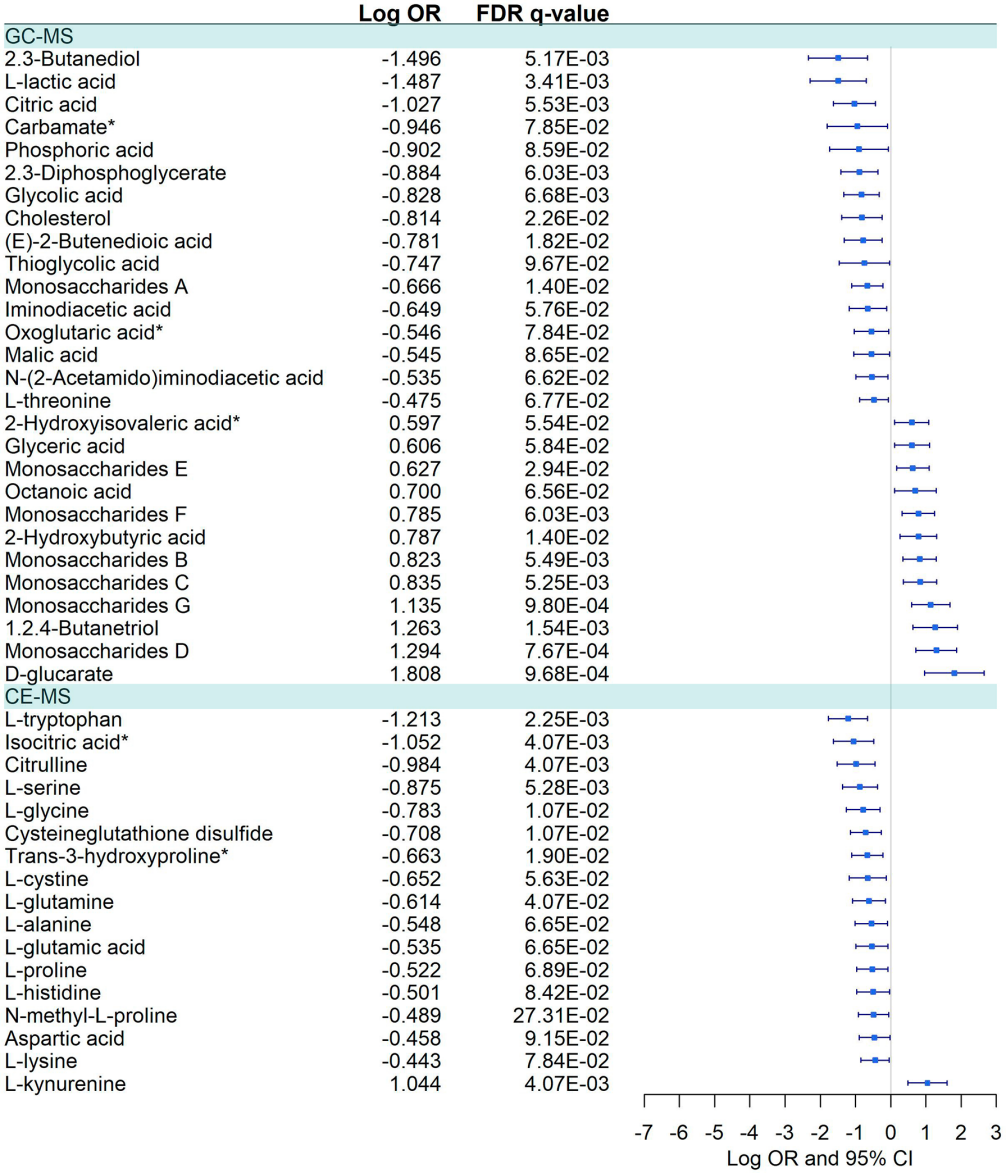
Pairwise comparisons between Severe vs. Moderate patients in GC-MS and CE-MS metabolites. Multivariate logistic regression, adjusted for age and sex, was used to compare severe vs. moderate patients. The False Discovery Rate or FDR was used to cope with multiple testing, q-values are provided. Carbamate*: Tris(trimethylsilyl)carbamate. Oxoglutaric acid*, Alpha-ketoglutaric acid. 2-Hydroxyisovaleric acid*, 2-Hydroxy-3-methylbutyric acid/Pentanoic acid 2-[(trimethylsilyl)oxy]-, trimethylsilyl ester. Trans-3-hydroxyproline*, trans-3-hydroxyproline/trans-4-hydroxyproline/cis-4-Hydroxy-D-proline. Isocitric Acid*, Citric Acid/Isocitric acid. D-Alanyl-D-Valine*, D-Alanyl-D-Valine/Glycyl-L-leucine/N2-Acetyl-L-Lysine.

Forty-five metabolites were dysregulated, 28 in GC-MS and 17 in CE-MS (**Figure 6**), mainly amino acids, fatty acids, and monosaccharides. About half of the metabolites in GC-MS were down-regulated and the other half upregulated in severe COVID-19, while in CE-MS, most of the metabolites were down-regulated. A total of eight metabolic pathways were enriched using the significant 45 metabolites. Two of those pathways were common to all previous comparisons, and four of them were previously dysregulated in COVID-19 patients vs. healthy controls (**Figure 4**; **Supplementary Data 10**).

#### Dysregulated Metabolites Across Pairwise Comparisons

Sixteen metabolites were dysregulated between COVID-19 patients vs. healthy controls and symptomatic vs. asymptomatic/mild comparisons (**Supplementary Data 11**). However, 13 metabolites were differentially dysregulated in both comparisons (glycerol monostearate, monostearin, L-cysteine, glyceraldehyde, myristic acid, D-alanyl-D-valine, monosaccharides F, citric acid, D-glucarate, L-tryptophan, L-proline, L-alanine, and L-histidine) (**Figures 2** **–** **5**; **Supplementary Data 11**).

COVID-19 patients vs. healthy controls and severe vs. moderate pairwise comparisons shared the largest number of dysregulated metabolites, 22 in GC-MS and 13 in CE-MS (**Supplementary Data 11**). However, most of them presented differential dysregulation (positive or negative dysregulation) between both pairwise comparisons.

Differential dysregulation was also found in six metabolites (monosaccharides E, monosaccharides A, cholesterol, D-glucarate, cysteineglutathione disulfide, and L-cystine) in severe vs. moderate and symptomatic vs. asymptomatic/mild pairwise comparisons for GC-MS and CE-MS (**Supplementary Data 11**).

### Plasma Biomarkers Levels Correlated With Dysregulated Metabolites

**Figure 7:**
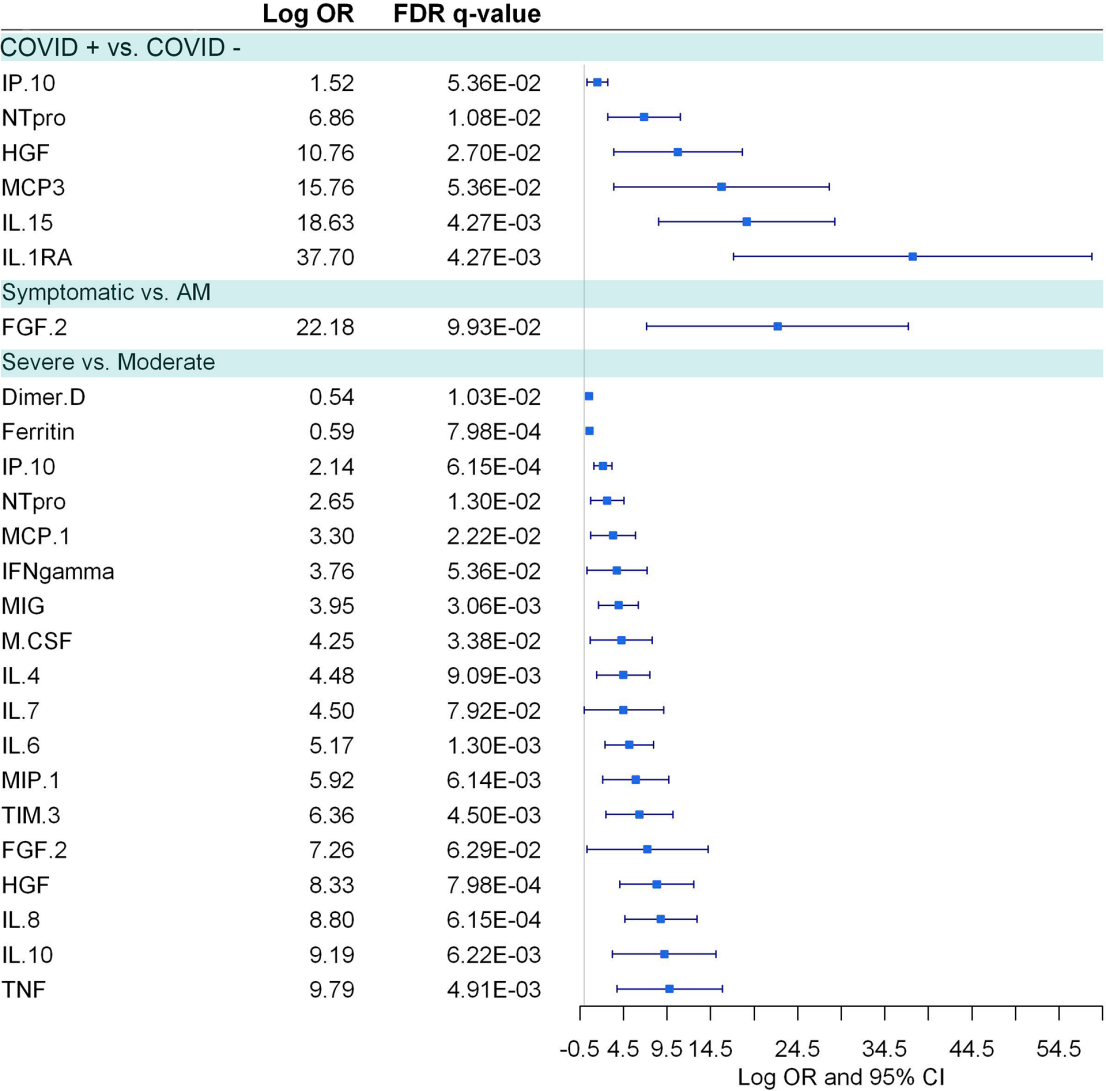
Pairwise group comparisons of cytokines. Logistic regression, adjusted for age and sex, was used. The False Discovery Rate or FDR was used to cope with multiple testing, q-values are provided.

COVID-19 patients, in comparison to healthy controls, showed six elevated biomarkers (IP-10, NT-proBNP, HGF, MCP-3, IL-15, IL-1RA) (**Figure 7**). Besides, 16 plasma biomarkers showed significant correlations (FDR<0.1) with dysregulated metabolites in COVID-19 patients (**Supplemental Data 12**). Two metabolites (L-kynurenine and L-tryptophan) were the metabolites that correlated with more cytokines, especially with pro-inflammatory cytokines (IL-4, IL-6, IL-8, IP-10, TNF alpha, and HGF), but also with an anti-inflammatory cytokine (IL-10). L-kynurenine correlated positively and L-tryptophan negatively with all the mentioned cytokines, respectively, which shows the close relationship between the L-kynurenine/L-tryptophan ratio and the inflammatory process in COVID-19 disease. Among the pro-inflammatory cytokines, IL-6 and IP-10 were the ones that correlated with more metabolites. Interestingly IP-10 negatively correlated with many amino acids and positively with phenylalanine (**Supplementary Data 12**).

Symptomatic COVID-19 patients, in comparison to asymptomatic/mild individuals, had only FGF-2 elevated (**Figure 7**). Besides, 11 inflammatory biomarkers were significantly correlated with 19 deregulated metabolites in symptomatic COVID-19 patients (**Supplementary Data 13**). As described above, L-tryptophan and L-kynurenine were negatively and positively correlated, respectively, with different pro-inflammatory cytokines like IL-6, IL-8 IP-10, TNF-α, and TIM-3.

Severe COVID-19 patients, in comparison to moderate individuals, showed 18 plasma biomarkers (D-dimer, ferritin, IP-10, NT-proBNP, MCP1, IFN-γ, MIG, M-CSF, IL-4, IL-7, IL-6, MIP-1, FGF-2, TIM3, HGF, IL-8, IL-10, and TNF-α.), 12 of those pro-inflammatory biomarkers (**Figure 7**). Of them, four plasma biomarkers were correlated with ten dysregulated metabolites in moderate COVID-19 patients (**Supplementary Data 14**), and no significant correlations were found within the severe COVID-19 cases.

### Sex-Specific Metabolites and Cytokine Dysregulation

Interaction with sex was significant in 29 (27.6%) metabolites (21 GC-MS and 8 CE-MS) and 19 (73%) plasma biomarkers, suggesting that both sexes have a differential dysregulation for these molecules (**Supplementary Data 15, 16**).

**Figure 8:**
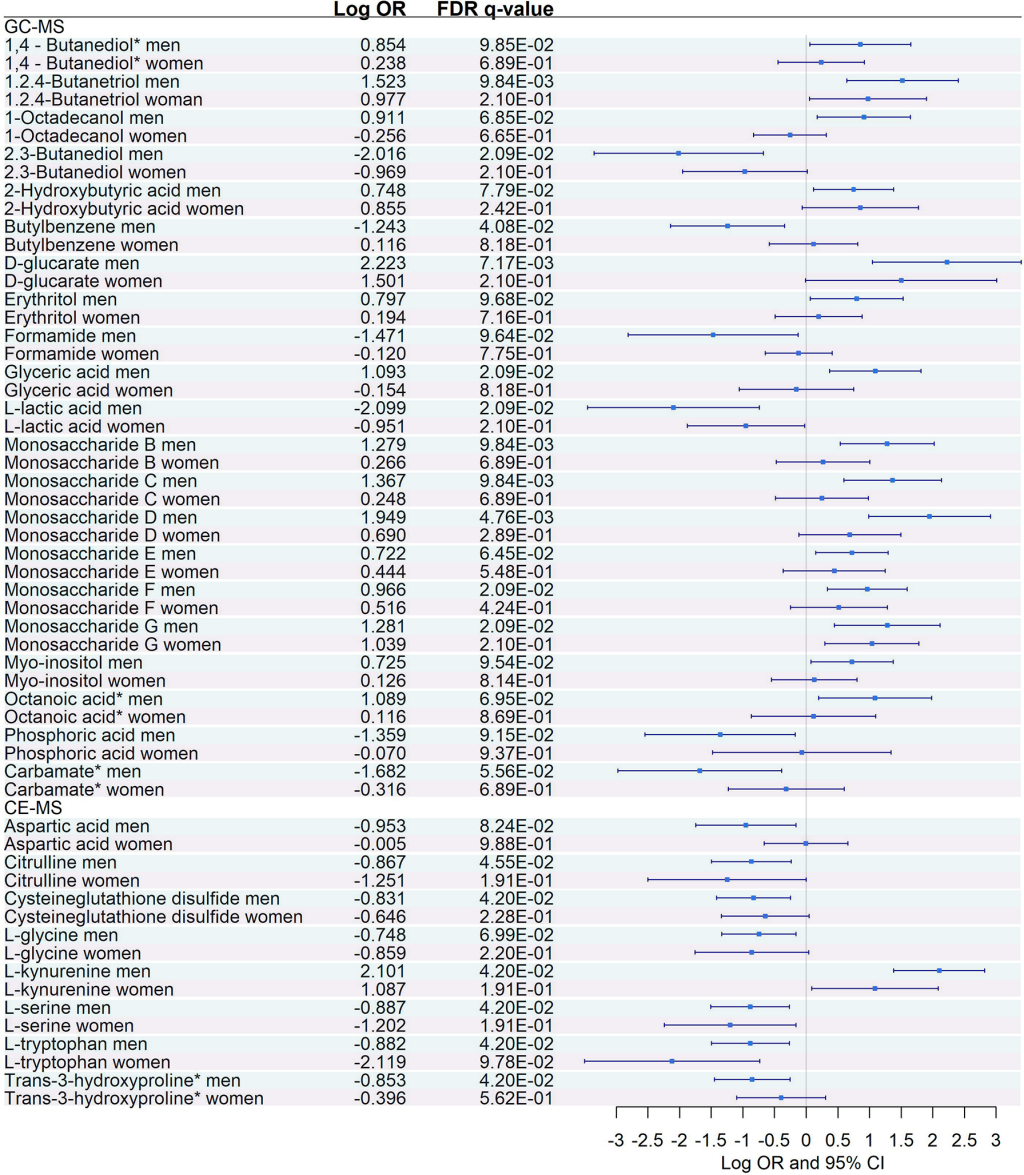
Sex-specific pairwise comparisons between Severe vs. Moderate patients in GC-MS and CE-MS metabolites Logistic regression, adjusted for age, was used for each sex separately. The False Discovery Rate or FDR was used to cope with multiple testing, *q*-values are provided. 1,4-Butanediol*, (R*,S*)- 3,8-Dioxa-2,9-disiladecane, 2,2,9,9-tetramethyl-5,6-bis[(trimethylsilyl)oxy]. Carbamate*, Tris(trimethylsilyl)carbamate. Trans-3-hydroxyproline*, trans-3-hydroxyproline/trans-4-hydroxyproline/cis-4-Hydroxy-D-proline.

**Figure 9:**
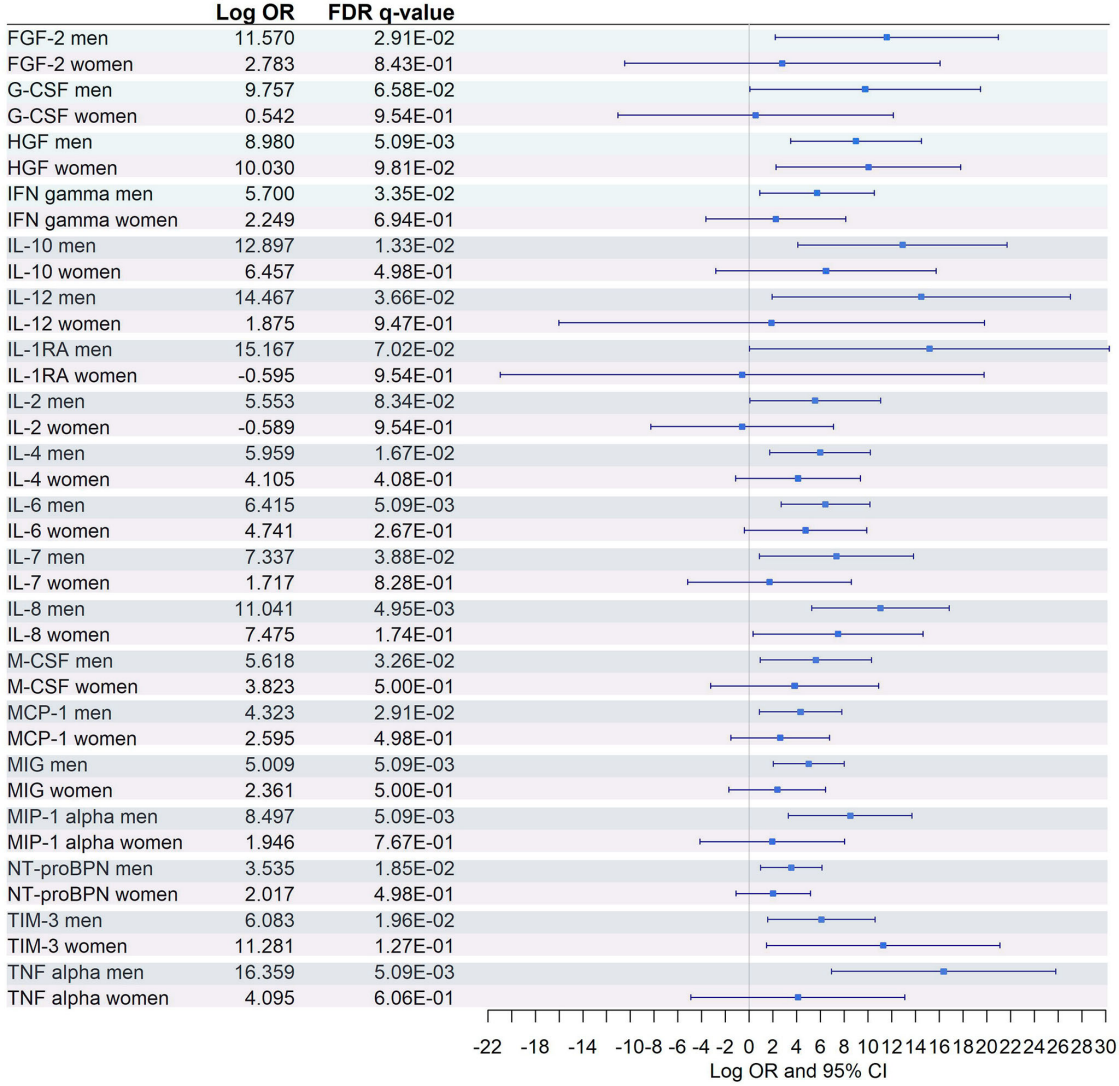
Sex-specific pairwise comparison of cytokines. Logistic regression, adjusted for age, was used for each sex separately. The False Discovery Rate or FDR was used to cope with multiple testing, q-values are provided.

Logistic regressions between severe and moderate COVID-19 were performed independently for each sex, adjusting by age (**Figures 8**, **9**). Among the 21 GC-MS metabolites that showed a significant interaction with sex, no significant dysregulation was found for women (after correcting for multiple testing using FDR) (**Figure 8**), while all of them were significantly dysregulated in men.

Similarly, among the 8 CE-MS metabolites that showed significant interaction with sex, only L-tryptophan showed a significant dysregulation and a more prominent effect (logOR in men = -0.88, logOR in women = -2.12) between women with severe and moderate COVID-19 (**Figure 8**). This trend, characterized by a lack of significant dysregulation in women between moderate and severe patients, was also found among plasma biomarkers that showed significant interaction with sex (**Figure 9**). After multiple corrections with FDR (cut-off in *q*-value < 0.1), only HGF was significantly dysregulated in women (**Figure 9**).

## Discussion

Our study provides a non-targeted metabolomics characterization of COVID-19 infection and severity at disease onset, while previous studies showed high heterogeneity regarding sampling timing (8, 10, 14, 15, 26, 27). In addition, to our knowledge, this study shows one of the first sex-specific dysregulation in both metabolite and cytokine levels between moderate and severe cases prior to the moment of worst evolution.

### COVID-19 Metabolic Characterization

Our results showed that pathways linked to amino acids and their intermediates, nucleotide metabolism, and energy metabolism were upregulated in patients with acute respiratory distress syndrome (ARDS) with respect to healthy controls, which is consistent with previous reports (28). Moreover, we also found an upregulation of L-tryptophan and L-kynurenine in COVID-19 patients, also showed in previous studies (9, 13, 15). L-tryptophan is a metabolic node where many different pathways are gathered, like the regulation of angiotensin-converting enzyme 2 (ACE2), mammalian target of rapamycin (mTOR) activation, both related to COVID-19, and cell proliferation and survival (16). L-kynurenine is a mediator of tryptophan degradation pathway, where several immunomodulatory metabolites are produced. Tradditionally, disruption of this pathway has been related to neuropsychiatric and neurodegenerative disorders, but COVID-19 also disrupts this pathway (9). Additionally, energy and oxygen consumption metabolism was upregulated in COVID-19, which is probably due to the metabolic changes observed in COVID-19 patients, as the acidosis caused by hypoxic conditions. In this setting, we detected increased levels of xanthotoxin, an epoxide intermediate, closely related to cytochrome P450 (CYP450) family enzymes that mediates oxidation of xenobiotics to more polar species. However, in epoxide situations, CYP450 catalysis can generate reactive species, like xanthotoxin, that covalently bind to and inactivate the CYP450 responsible for their formation and bind to other cellular proteins that may culminate in cell death (29). Under hypoxia, levels of pyruvate and lactic acid are increased while the flux of acetyl-CoA is inhibited, showing a potential inhibition of the Krebs cycle. Additionally, the altered levels of citrate may be explained by the ATP citrate lyase activity. This dysregulation of the Krebs cycle is also supported by the abundance of ketone bodies and monosaccharides due to glycolysis in the absence of oxygen. An upregulation of L-carnitine, and its ester form L-acetylcarnitine in COVID-19 patients is (9) one example of this metabolic dysregulation.

Additionally, we explored the relationship between metabolites and plasma inflammatory markers as metabolism and the inflammatory response are closely related (30). COVID-19 patients showed higher levels of pro-inflammatory cytokines, such as IP-10 IL-15,IL-1RA NT-proBNP, and the angiogenic growth factor HGF, among others. This pro-inflammatory status activate the kynurenine pathway, as we observed that the higher level of L-kynurenine was positively correlated with most of these pro-inflammatory markers such as NT-proBNP, IP-10 and TIM-3. The disruption of kynurenine pathway could allow SARS-CoV-2 to evade immune response (9), weakening the immune system.

Lactic acid has been also involved in inflammatory processes by inhibiting the motility of CD8^+^ T cells, and it has been shown an anti-inflammatory effect on cytokine production (31). *In vitro* assays have shown that lactic acid reduce LPS-induced IL-1, IL-6, and TNF-α production and increase IL-10 (31). Hence, our study supports these findings as it reports significant negative associations of lactic acid with IL-8, IL-6, and IP-10 cytokines. Additionally, iminodiacetic acid, a metabolite that has been useful as a biomarker to predict ARDS severity (28), has a positive correlation with IP-10. On the other hand, IL-10 is the only anti-inflammatory cytokine correlated with several metabolites. Besides a negative correlation with L-tryptophan, IL-10 also shows interesting patterns with other metabolites. It correlated positively with carnitine which seems to be an anti-inflammatory agent by preventing oxidative damage by reactive oxygen species (ROS) (32, 33). By reducing lipid peroxidation, carnitine increases antioxidant defense through antioxidant enzymes. IL-10 also has a significant positive correlation with another antioxidant metabolite, 2-aminobutyric acid (2-AB), through the maintenance of the reduced glutathione (GSH) levels (34), and it is an antioxidant by itself, preventing ROS production and anti-inflammatory cytokines by macrophages (35). Interestingly, the upregulation of IL-10 is correlated with the upregulation of hypoxia-associated metabolites like phenylalanine and iminodiacetic acid.

### Metabolic Profile of Symptomatic vs. Asymptomatic/Mild Patients

Symptomatic status was associated to a substantial reduction of citric and isocitric acid, and a mild downregulation of D-glucarate and other pyruvate amino acid precursors (L-glycine, L-alanine, L-cysteine, and L-threonine). This effect is reinforced by changes in the carbohydrate metabolism, as the reduction of D-glyceraldehyde and different monosaccharides, due to changes in the glycolysis/gluconeogenesis balance. The urea cycle is a downregulated pathway under hypoxia conditions (9, 16, 26). Thus, reduced citrulline levels or increased L-phenylalanine levels could be a consequence of the hypoxia provoked by COVID-19 in symptomatic individuals. Interestingly, phenylalanine positively correlates at the same time with IP-10 (proinflammatory) and with an anti-inflammatory one, IL-10. Instead, citrulline negatively correlates with IP-10 and positively with NT-proBNP. Our results show a complex regulation of fatty acids pathways with downregulation of azelaic acid and myristic acids, but upregulation of cholesterol or monostearin (28), which is in line with previous studies (6, 9, 11, 16).

Additionally, we observed a downregulation of L-tryptophan in symptomatic COVID-19 patients, as previously was described in COVD-19. There is limited evidence for the L-tryptophan role in inflammation, but it has been linked to several age-related diseases (36). Activation of L-tryptophan metabolism and the L-kynurenine pathway have been linked to the prevention of hyperinflammation. However, downregulation of L-tryptophan could provoke chronic inflammation or inflammaging, which is a significant risk factor for morbidity and mortality, as it causes defects in components of the innate and adaptive immune systems leading to decreased immune responses with age as well as increased severity of infections (37).

In addition, L-tryptophan catabolism into L-kynurenine by indoleamine 2,3-dioxygenase (IDO) is linked to Treg/Th17 differentiation and immune activation. Tregs are a subset of T cells modulating the function of several immune cells to promote immune tolerance and maintain intestinal immune homeostasis (38). L-tryptophan metabolism can induce the differentiation of Tregs through kynurenine pathway and microbiota-mediated degradation. In patients with severe COVID-19, decreased Treg and increase in Th17 cells have been reported, showing an insufficient regulation of pro-inflammatory immune response that may further aggravate inflammatory response, production of cytokines, worsening tissue damage and leading to multiorgan failure and death (39). In addition, L-tryptophan act as a central hub for host/microbial symbiosis which is not restricted to the gut and could be modulating the microbiome of other body sites, such as the lungs. Studies in COVID-19 patients have described that in addition to intestinal dysbiosis, pharyngeal and pulmonary microbiota were also unbalanced, evidencing that there is a close crosstalk between gut and lung microbiota (40). In this regards, either gut and lung microbiome, are probably influencing tryptophan metabolism and kynurenine pathway, promoting local and systemic responses to control inflammation (41).

Similarly to previous section, we also report a strong correlation of L-tryptophan and L-kynurenine with different cytokines. Interestingly, we found that the fibroblast growth factor (FGF2), involved in angiogenesis events and viral infections is upregulated in symptomatic patients, as previously described (42), but its regulation is unclear (43). FGF2 is also disrupted in influenza A (H1N1) infection,Middle East respiratory syndrome coronavirus (MERS-CoV) infection (44),

Among symptomatic patients, we also highlight the positive correlation found between NT-proBNP and L-kynurenine, citrulline, L-cysteine, glycerol monostearate, methyl stearate and monostearin, and negative correlation with L-tryptophan. Many of these correlations were also found in the COVID-19 infected group. In this setting, these metabolites could be involved in cardiac injury, a common complication of COVID-19, as it has been widely described for elevated levels of NT-proBNP (45). Similarly, circulating HGF has been also identified as a prognostic marker of severity (46) and cardiac injury (47) and it was found positive and negatively correlated with L-kynurenine and L-tryptophan. In addition, citric acid, a metabolite with anti-inflammatory activity, antiplatelet aggregation and direct cardiomyocyte protective effects (48) was found to be the most downregulated metabolite among symptomatic patients. Its reduced levels correlated with elevated levels of thrombosis markers (D-dimer), acute phase reactants (ferritin), and inflammatory modulators (HGF and IP-10), suggesting that strategies that increase citric acid levels could be protective for COVID-19 infected patients.

### Progression to Severe COVID-19 (Severe vs. Moderate)

We observed a complex dysregulation of amino acid metabolism in severe patients. Glycogenic (L-alanine, L-histidine, and L-glucarate) and sulfur-containing amino acids (L-cysteine) are downregulated, while oxidized forms of sulfur-containing amino acids (L-cystine and cysteineglutathione disulfide) are upregulated, among others. Alterations of the aminoacidic metabolism are generalized in severe COVID-19 patients, including pathways of multiple amino acids, nitrogen metabolism, and aminoacyl-tRNA synthetase (ARS) (8, 11, 26), which are mainly derived from lung damage and hypoxic conditions. Dysregulation of the nitrogen metabolism is also accompanied by significant alterations of the carbon metabolism (9, 49, 50), which might resemble a metabolism alteration in liver. Amino acids degradation and ammonium ions convertion into urea is mainly produced in the liver, whose dysfunction has been described in patients with severe viral diseases, possibly due to direct infection or as part of the systemic inflammation.

The hepatic urea cycle is the key detoxification metabolic pathway, and urea cycle related metabolites such as citrulline and aspartic acid, are also downregulated, in line with previous studies (6, 9). During amino acid degradation, the urea cycle requires two amino groups, one of which is provided by the aspartic acid. Under hypoxic conditions, the condensation reaction between the amino group of aspartate and the carbonyl group of the citrulline is affected, being reflective of metabolic disturbances in the urea circulation and the nitrogen metabolism (9, 10, 15, 16). Additionally, we also observed a deep disruption of the nitrogen-related cycle ARS pathway, which is involved in loading transfer RNAs (tRNAs) with their cognate amino acids (51, 52), being essential for protein translation. Nevertheless, a growing body of evidence suggests that ARS is involved in the immune response to viral infection, essential in HIV-1, Japanese encephalitis virus, West Nile virus and gastroenteritis coronavirus infections, among others (52). ARS, specially mitochondrial ARS (mtARS), has recently been identify as a key role in severe COVID-19, describing a direct interaction between SARS-CoV-2 and several mtARS (53).

Furthermore, some of the most dysregulated metabolites were the L-tryptophan and L-kynurenine, which constitute a potent immunomodulatory pathway opposing hyperinflammatory responses. L-tryptophan degradation generates kynurenines, which have immunoactive effects. The L-kynurenine/L-tryptophan ratio has been extensively studied in inflammatory diseases since was introduced decades ago as an index of tryptophan breakdown (54). In the context of COVID-19 infection, this ratio has also been explored (9, 13, 15) as it is related to the inflammatory state in COVID-19, and can be used in treatment decision (55), with some studies describing an increase in the L-kynurenine/L-tryptophan ratio associated with COVID-19 severity (55, 56), poor prognosis (57, 58),) and mortality (58–60),,. Consistently with these studies, we observed an upregulation of L-kynurenine and downregulation of L-tryptophan in severe COVID-19 patients, indicating an elevated L-kynurenine/L-tryptophan ratio, which suggests a disease-associated hyperactivation of the indoleamine-pyrrole 2,3-dioxygenase (IDO) enzyme. IDO leads the conversion of L-tryptophan into L-kynurenine in immune cells, which is strongly activated in response to interferons and inflammatory cytokines released upon inflammation (61). In this setting, L-kynurenine will increase the activation of the aryl hydrocarbon receptor (AhR), a ligand-activated transcription factor widely expressed by immune cells and with a key role in the host response to viral infections and gut immune homeostasis (62).This activation in T-effector cells promotes the transformation into Treg cells and IDO induction, which maintains immunosuppression (63). AhR activation also contributes to dysregulate the initial production of pro-inflammatory cytokines during SARS-CoV-2 infection, leading to an increase in virus entry into a number of cells. In this setting, an association between AhR expression and viral load in SARS-CoV-2 infected patients has been detected (64). Besides, AhR activation on NK and CD8+T leads to a state of exhaustion that limits their capacity to eliminate virus-infected cells, whilst increasing the activation of the early-activated immune cells, such as macrophages (65). Additionally, AhR signaling pathway contributes to the lung pathogenesis associated with SARS-CoV-2 infection, as may interfere with lung epithelial barrier integrity. Indeed, the triggering of AhR signaling by SARS-CoV-2 leads to the overexpression of mucins, limiting the O2 diffusion and therefore hampering lung pathology (64). In this regard, pharmacological inhibition of AhR has been proposed as therapy to increase the host’s antiviral response and consequently reduce viral replication, as well as reducing the mucins expression and limiting lung pathology during COVID-19 (66).In addition, it is important to point out that some metabolites showed differential deregulation in different comparisons. For example, elevated levels of L-glycine, L-serine, L-alanine, L-tryptophan, malic acid, citric acid, L-proline, L-glutamic acid, L-lactic acid among others were found in COVID-19 infected patients compared to healthy individuals. In contrast, such metabolites were found to be significantly decreased at presentation in patients who followed a severe course of the disease in comparison with moderate patients. This could indicate that in the early stages of infection, COVID-19 causes an increase of a large number of metabolites and metabolic pathways, even in patients who will not experience significant disease. However, patients who will develop a severe disease could show an early difficulty in activating certain metabolic pathways compared to patients with a moderate course of the disease. Similar differential findings across the diverse severity groups, at disease onset, have been previously described for other biomarkers (67).

### Sex-Specific Effects in Progression to Severe COVID-19

We have identified an elevated number of metabolites, cytokines, and chemokines with strong interaction with sex. To our knowledge, this is the first time that sex-specific metabolic dysregulation has been tested and observed when comparing moderate and severe COVID-19 patients. Sex-specific metabolites are directly related to energy metabolisms, like monosaccharides, glyceric acid, or erythritol, synthesized directly from glucose *via* the pentose phosphate pathway (68). Furthermore, other energy-related metabolites were found to interact with sex: glucuronic acid derivatives, like D-glucarate, ketone bodies regulators, like octanoic acid (69), or even predictors of dysglycemia such as 2-hydroxybutyric (70). We also found keystone metabolites involved in many different processes, like lactic acid, related to immunological processes but also to energy and nitrogen metabolism dysregulation. Sex-specific effect in nitrogen metabolism was detected through multiple metabolites like aspartic acid, citrulline, cysteineglutathione disulfide, L-glycine, L-serine, and formamide. We also found other metabolites related to oxidation and ROS production in human neutrophil granulocytes like butylbenzene (71). Finally, we also found a differential alteration of metabolites with documented interactions with cytokines such as myo-inositol, which downregulates IL-6 expression and is also the precursor of phospholipids present in the surfactant (72); lactic acid, which has been widely involved in inflammatory processes (73); serine, which is considered a crucial immune metabolite that modulates adaptive immunity (74); citrulline, which is a precursor of arginine and improve macrophage function in mammals (75) or L-kynurenine, which has been positively correlated with diverse cytokines, among others. For all these metabolites related to different cellular functions, significant dysregulation was found mainly in men, not in women. Exceptions to this are L-serine, L-kynurenine, and L-tryptophan, which showed dysregulation both in men and in women. To our knowledge, scarce literature has been published about sex-differences in metabolomic profiles in COVID-19. Recently, it has been described that males had higher levels of both L-tryptophan and L-kynurenine (76), which is in concordance with our results, where we found a higher increase of L-kynurenine and a less prominent decrease of L-tryptophan in males. Similarly, a higher L-kynurenine/L-tryptophan ratio has been described in males (55) (77), which has been related to increased levels of proinflammatory cytokines/chemokines and poorer outcomes. Therefore, L-kynurenine metabolism seem to be implicated in sex-specific immune response in COVID-19 (77), possibly by activation of AhR. However, further studies are needed to address the role of L-kynurenine and the L-kynurenine/L-tryptophan ratio in sex-specific differences in COVID-19, since men have a higher risk for COVID-19 infection, hospitalization, disease severity, ICU admission and death (78).

Nineteen out of 26 cytokines were identified to have significant interaction with sex. Interestingly, just one (HGF) was found to be dysregulated between severe and moderate COVID-19 patients in both men and women. For the remaining inflammatory markers, significant dysregulation was found only in men.

### Limitations and Strengths

Our study had a limited sample size and an uneven number of patients among the different COVID-19 severity groups. This limitation prevented us from assessing metabolomic sex-specific effects between COVID-19 patients and healthy individuals or between symptomatic and asymptomatic/mild patients. However, it is important to note that this study has a large sample size compared to those published to date. Besides, about half of the features could be identified. This is the standard level of identification in metabolomics analysis since some signals do not correspond to actual metabolites, not all potential metabolites are known due to the limitations of current metabolomics databases, and some signals correspond to peptides, which are not considered. Additionally, our cohort shows high homogeneity, enabling us to obtain meaningful comparisons among different COVID-19 severity groups. No effect of age, body mass index or other comorbidities were found between groups. Also, all samples were obtained at disease onset or hospital admission, which provides valuable knowledge about altered metabolic pathways before the highest severity status of the disease. Finally, our study provides a relevant characterization of the metabolomic profile of COVID-19 patients in early waves of COVID-19 originated by the original variant. Therefore, our results are not biased by previous infection, reinfection or vaccination status. Further studies comparing the metabolomic outcomes of patients infected by different variants of concern would also be of interest to decipher possible changes in the underlying pathophysiology in different waves of infection.

### Conclusions

In conclusion, a complex metabolomic systemic dysregulation was observed between different COVID-19 severity groups from the early COVID-19 stages. An increased transformation of L-tryptophan into L-kynurenine and a strong dysregulation in many other amino acids’ pathways and their intermediates, nucleotide metabolism, energy metabolism, and nitrogen metabolism were found, along with changes in the levels of immune-related markers. These findings open new perspectives to enhanced knowledge of the molecular mechanism that underpins host-virus interaction in SARS-CoV2 infection, which is critical to both clinical management and to improve diagnostic tools that can stratify patients into risk categories or require urgent intervention.

## Data Availability Statement

The data presented in the study are deposited in the metabolomics workbench repository (https://www.metabolomicsworkbench.org/data/DRCCDataDeposit.php), accession number ST002194 (http://dx.doi.org/10.21228/M8141Z).

## Ethics Statement

The studies involving human participants were reviewed and approved by Committee of the Institute of Health Carlos III (PI 33_2020-v3). The patients/participants provided their written informed consent to participate in this study. Written informed consent was obtained from the individual(s) for the publication of any potentially identifiable images or data included in this article.

## Author Contributions

Funding body and study concept design: AF-R and MJ-S. Patients’ selection and clinical data acquisition: PR, OMG, FP-G, MM-V, RB, JC-G, NB-L, and IRM-A. Sample preparation and plasma biomarker analysis: MM-V, AV-B, EV-A, SB-S, OB-K, OA-D, and CB. Statistical analysis and interpretation of data: FC, AV-B, AF-R, and MJ-S. Drafting of the manuscript: FC, AF-R, and MJ-S. Critical revision of the manuscript for relevant intellectual content: OA-D, CB, PR, RB, FP-G, OM-G, IM, and SR. Supervision: AF-R and MJ-S. All authors read and approved the final manuscript.

## Funding

This study was supported by grants from Instituto de Salud Carlos III (ISCIII; grant number COV20/1144 (MPY224/20) to AF-R/MJ-S). The study was also funded by CIBER - Consorcio Centro de Investigación Biomédica en Red - (CB 2021; CB21/13/00044), Instituto de Salud Carlos III, Ministerio de Ciencia e Innovación and Unión Europea - NextGenerationEU. AF-R and MJ-S are Miguel Servet researchers supported and funded by ISCIII (grant numbers: CP14CIII/00010 to AFR and CP17CIII/00007 to MJ-S). Universidad Alfonso X el Sabio, grant number 1.013.005

## Conflict of Interest

The authors declare that the research was conducted in the absence of any commercial or financial relationships that could be construed as a potential conflict of interest.

## Publisher’s Note

All claims expressed in this article are solely those of the authors and do not necessarily represent those of their affiliated organizations, or those of the publisher, the editors and the reviewers. Any product that may be evaluated in this article, or claim that may be made by its manufacturer, is not guaranteed or endorsed by the publisher.

## Abbreviations

ARDS, Acute respiratory distress syndrome; ARS, aminoacyl-tRNA biosynthesis; ACE2, angiotensin-converting enzyme 2; CE-MS, capillary electrophoresis-mass spectrometry; CV, coefficient of variation; CYP450, cytochrome P450; eCRF, electronic case report form; FDR, false discovery rate; FGF2, fibroblast growth factor; FI, fluorescence intensity; IDO, indoleamine-pyrrole 2,3-dioxygenase; LOOCV, leave-one-out cross-validation; GC-MS, like gas chromatography-mass spectrometry; mTOR, mammalian target of rapamycin; MERS-CoV, Middle East respiratory syndrome coronavirus; MFE, molecular feature extraction; PLS-DA, partial least squares - discriminant analysis; VIP, Variable importance in projection; ROS, reactive oxygen species; GSH, reduced glutathione.
