# Supplementary material for: Metabolic Profiling at COVID-19 Onset Shows Disease Severity and Sex-Specific Dysregulation
Source: Front Immunol. 2022 Jun 30;13:925558. doi: 10.3389/fimmu.2022.925558 (PMC9280146; doi:10.3389/fimmu.2022.925558)

**Supplementary Data 1.** Flowchart describing the classification of patients concerning COVID-19 severity. **Abbreviations:** ICU. Intensive care unit; ARDS, acute respiratory distress syndrome; AM: asymptomatic/mild patients**.**



**
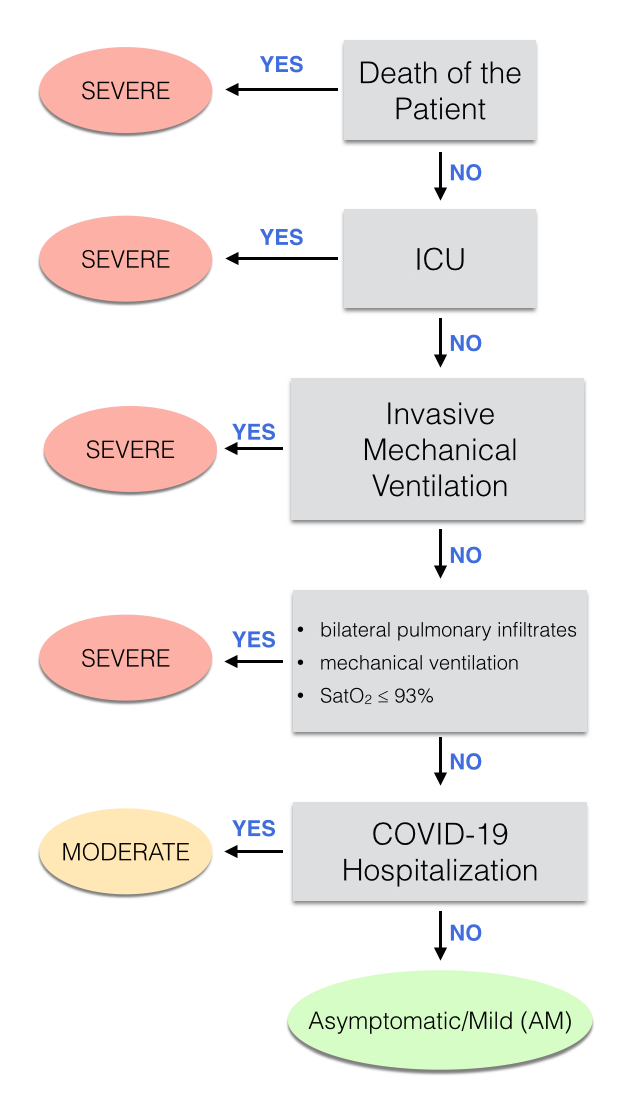
**

**Supplementary Data 2: Additional description of non-targeted metabolomics method**

**Sample treatment**

Plasma samples were inactivated with cold (-20 °C) MeOH:EtOH (1:1, v/v). Samples were vortex-mixed for 1 min, incubated on ice for 5 min and centrifuged for 20 min at 16000 *xg* at 4 °C. The resulting supernatant was stored at -80 °C until analysis by gas chromatography-mass spectrometry (GC-MS) and capillary electrophoresis-mass spectrometry (CE-MS). Sample preparation for GC-MS and CE-MS was carried out at CEMBIO (Madrid, Spain) based on previously developed methods for plasma samples [1] [2].

For GC-MS analysis, 200 µL of frozen plasma supernatant was thawed at room temperature and 30 µL of 80 mg/L deuterated palmitic acid in MeOH was added as internal standard (IS). After samples were evaporated to dryness a two-step derivatization process was done. First, methoximation was performed by adding 20 µL of *O*-methoxyamine hydrochloride (15 mg/mL in pyridine) and vortex-mixing for 5 min. Then, 3 cycles of ultrasonication for 5 min and vortex for 5 min were done. Immediately after, vials were incubated in darkness at room temperature for 16 h. Second, silylation was carried out by adding 20 µL of BSTFA/TMCS (99:1) followed by vortex-mixing for 5 min. Then, capped vials were incubated in the oven at 70 °C for 1 h. Finally, 100 µL of heptane containing 20 mg/L of tricosane as IS was added to each vial prior to injection. For CE-MS analysis, 200 µL of frozen supernatant was thawed until room temperature and it was evaporated to dryness using a SpeedVac Concentrator System (Thermo Fisher Scientific, MA). Afterwards, 100 µL of 0.2 mM methionine sulfone (MetS) as internal standard (IS) in 0.1 M formic acid solution was added. Samples were vortex mixed for 1 min, transferred to a Millipore filter (30 kDa protein cutoff) and centrifuged for 40 min at 2000 x*g* at 4 °C. Finally, the filtrate was transferred to a CE-MS vial for analysis.

Quality control samples (QC) were prepared by adding equal volumes of plasma supernatant from each sample and were prepared as previously mentioned for GC-MS and CE-MS analysis. Blank solutions were also prepared with MeOH:EtOH (1:1, v/v). All samples were randomized during analysis.

**Non-targeted metabolomics**

Samples were analyzed by two different platforms: GC-MS, and CE-MS using previously developed methods and following the next analytical conditions [3].

An Agilent GC-MS system (8890) coupled to a single quadrupole mass spectrometer (5977B, Agilent Technologies) was used to analyze plasma samples. Two µL of derivatized samples were automatically injected in split mode (split ratio 1:10) by an Agilent autosampler (7693) into an Agilent ultra-inert deactivated glass wool split liner. Metabolite separation was carried out in a DB5-MS GC column (length, 30 m; inner diameter, 0.25 mm; and 0.25 µm film of 95% dimethyl / 5 % diphenylpolysiloxane), with a pre-column (10 m J&W integrated with Agilent 122-5532G). The flow rate of helium carrier gas was constant at 1.1359 mL/min and the injector temperature was set at 250 °C. The lock of the retention time (RTL) relative to the internal standard (methyl stearate) peak at 19.66 min was performed. The oven temperature gradient was initially set at 60 °C and was maintained for 1 min. Then it was raised by 10 °C/min until it reached 325 °C, and then was held at this temperature for 10 min before cooling down. The total analysis run time was 37.5 min. The transfer line temperature was stablished at 280 °C and the electron ionization (EI) source was operated at 70 eV and the filament source temperature was set at 200 °C. Mass spectra were collected over a mass range of 50 - 600 *m/z* at a scan rate of 2.7 scans/s. Data were acquired using the Agilent MassHunter Workstation GC/MS Data Acquisition (version 10.0).

For CE-MS analysis, prepared plasma samples were analyzed in a 7100 capillary electrophoresis (CE) system coupled to a 6230 time-of-flight mass spectrometer (TOF-MS) and equipped with an electrospray ionization (ESI) source from Agilent Technologies. Metabolite separation was performed in a fused silica capillary (100 cm; inner diameter, 50 µm, Agilent Technologies). Before each analysis, background electrolyte (BFE) (0.8 M formic acid solution in 10 % MeOH; v/v) was flushed for 5 min (950 mbar). Samples injections were performed over 50 s at 50 mbar and BGE was injected after each injection for 10 s at 100 mbar to improve reproducibility. The separation was carried out with an internal pressure of 25 mbar and 30 kV voltage with a total analytical run time of 35 min. Data were acquired in positive ionization polarity with a full scan range from 70 to 1000 *m/z* at a rate of 1.36 scan/s. The rest of MS conditions were: fragmentor set to 125 V, skimmer to 65 V, OCT RF Vpp to 750 V, drying gas temperature to 200 °C, flow rate to 10 L/min, nebulizer to 10 psig, and capillary voltage to 3500 V. The sheath liquid used for detection contained two reference masses (5 µL of purine with *m/z* 121.0509 and 5 µL of HP-0921 with *m/z* 922.0098) in MeOH/water (1/1; v/v) with 1 mM formic acid and the flow rate was set to 0.6 mL/min (split 1:100). The data acquisition was made using the Agilent MassHunter Workstation (Agilent Technologies).

QC sample was regularly analyzed through the run to assess the data quality, analytical system stability and sample treatment reproducibility. Also, a pair of blank solutions were analyzed at the beginning and at the end of each analytical sequence.

**Data treatment**

The first step was to assess the analytical performance, the quality of the chromatograms and the IS signal reproducibility by analyzing the total ion chromatogram (TIC) obtained for each sample, QC samples, blanks and internal standard peaks.

Once TICs were checked, for GC-MS data treatment, raw data files were imported in the MassHunter Quantitative Unknown Analysis software to perform the deconvolution and identification of the metabolites by searching into two target libraries: Fiehn library (version 2008) and the ‘in-house’ CEMBIO spectral library. Then, data obtained in the Unknowns Analysis Tool were aligned in Agilent Mass Profiler Professional version 15.1 and exported into Agilent MassHunter Quantitative Analysis version 10.0 to assign target ions and obtain the compound abundances.

In the case of CE-MS, after checking data quality, raw data were processed with MassHunter Profiler software (version 10.0), applying the molecular feature extraction (MFE) to clean data background, unrelated ions and find coeluting adducts (+H^+^ Na^+^ and K^+^ in positive ionization and neutral loss of water). All features extracted by MFE were then aligned across all samples with the batch recursive feature extraction (RFE) algorithm included in the same software. RFE uses the mass and retention time (RT) information obtained by MFE to improve the quality of the target list. The continuously infused references masses (*m/z* 121.0509 and *m/z* 922.0098) and those features found in blanks were excluded from the final list.

In both, GC-MS and CE-MS analysis, the final matrix was imported in Microsoft Excell to remove metabolites with poor reproducibility (coefficient of variation (CV) in the QCs greater than 30 %) as well as those features not presented in 70 % of samples in at least one sample group, and in GC-MS the final concentration of each metabolite was also normalized according to the IS abundance. Finally, QC intensity drop was corrected and matrices were further used for the statistical analysis

**References**

1. Garcia A, Barbas C: **Gas chromatography-mass spectrometry (GC-MS)-based metabolomics**. *Methods Mol Biol* 2011, **708**:191-204.

2. Naz S, Garcia A, Rusak M, Barbas C: **Method development and validation for rat serum fingerprinting with CE-MS: application to ventilator-induced-lung-injury study**. *Anal Bioanal Chem* 2013, **405**(14):4849-4858.

3. Naz S, García A, Barbas C: **Multiplatform analytical methodology for metabolic fingerprinting of lung tissue**. *Anal Chem* 2013, **85**(22):10941-10948.

**Supplementary Data 3**: Custom Procartaplex multiplex immunoassay (Invitrogen) panel.

| **Target Name** | **Full description** |
| --- | --- |
| D-dimer | D-dimer |
| Ferritin | Ferritin |
| FGF-2 | Fibroblast growth factor 2 |
| G-CSF (CSF-3) | Colony-stimulating factor 3 |
| HGF | Hepatocyte growth factor |
| IFN gamma | Interferon-gamma |
| IL-1 beta | Interleukin-1 beta |
| IL-10 | Interleukin-10 |
| IL-12 / IL-23p40 | Interleukin-12 |
| IL-13 | Interleukin-13 |
| IL-15 | Interleukin-15 |
| IL-1RA | Interleukin-1 receptor antagonist |
| IL-2 | Interleukin-2 |
| IL-4 | Interleukin-4 |
| IL-6 | Interleukin-6 |
| IL-7 | Interleukin-7 |
| IL-8 (CXCL8) | Interleukin-8 |
| IP-10 (CXCL10) | C-X-C motif chemokine ligand 10 |
| M-CSF (CSF1) | Colony-stimulating factor 1 |
| MCP-1 (CCL2) | C-C motif chemokine ligand 2 |
| MCP-3 (CCL7) | C-C motif chemokine ligand 7 |
| MIG (CXCL9) | C-X-C motif chemokine ligand 9 |
| MIP-1 alpha (CCL3) | C-C motif chemokine ligand 3 |
| NT-proBNP | N-terminal Pro-B type natriuretic peptide |
| TIM-3 (HAVCR2) | Hepatitis A virus cellular receptor 2 |
| TNF alpha | Tumor necrosis factor-alpha |

**Supplementary Data 4**: Demographic and comorbidities characteristics according to severity and biological sex. Numerator indicates the number of patients with available data, while denominator the total number of patients included in the study. Individual characteristics were summarized using standard descriptive statistics: mean ± standard deviation for continuous variables and count (percentage) for categorical variables. Differences between groups were tested using the Cochran-Mantel-Haenszel (C-M-H) test.

|  | Moderate | | Severe | |  | C-M-H test | |
| --- | --- | --- | --- | --- | --- | --- | --- |
|  | female | male | female | male |  | OR | p-val |
| **Demographics** |  |  |  |  |  |  |  |
| N | 30 | 34 | 14 | 33 |  |  |  |
| Age | 65.5±17.5 | 57.8±12.5 | 65.4±18.4 | 65.5±18.2 |  |  |  |
| BMI>=25 | 5/30 (16.7%) | 6/34 (17.6%) | 3/14 (21.4%) | 10/33 (30.3%) |  | 0.562 | 0.3306 |
| Smoke status (Yes) | 2/30 (6.7%) | 2/34 (5.8%) | 1/14 (7.1%) | 2/24 (6.1%) |  | 0.952 | 0.9522 |
| Former smoker | 2/30 ( 6.7%) | 6/34 (17.6%) | 3/14 (14.2%) | 8/33 (24.2%) |  | 0.521 | 0.3012 |
| **Comorbidities** |  |  |  |  |  |  |  |
| Hypertension | 14/30 (46.7%) | 13/34 (38.2%) | 7/14 (50%) | 16/33 (48.4%) |  | 0.686 | 0.4472 |
| Cardiopathy | 6/30 (20%) | 6/34 (17.6%) | 2/14 (14.3%) | 6/33 (18.9%) |  | 1.246 | 0.8671 |
| Chronic pulmonary disease | 1/30 (3.3%) | 3/34(5.9%) | 3/14 (21.4%) | 7/33 (21.2%) |  | 0.271 | 0.0652 |
| Chronic kidney disease | 2/30 (6.7%) | 2/34 (5.9%) | 2/14 (14.3%) | 7/33 (21.2%) |  | 0.286 | 0.0939 |
| Chronic liver disease | 1/30 (3.3%) | 2/34 (5.9%) | 0/14 (0%) | 2/33 (6.0%) |  | 1.301 | 0.7899 |
| Chronic neurological disease | 4/30 (13.3%) | 5/34 (14.7%) | 2/14 (14.3%) | 6/33 (18.9%) |  | 0.821 | 0.9261 |
| Neoplasia | 1/30 (3.3%) | 2/34 (5.9%) | 1/14(4.1%) | 3/33 (9.1%) |  | 0.569 | 0.7511 |
| Diabetes | 5/30 (16.7%) | 5/33 (14.7%) | 4/14 (28.6%) | 7/33 (21.2%) |  | 0.581 | 0.3934 |
| Chronic inflammatory disease | 1/30 (3.3%) | 1/33 (3.0%) | 2/14 (14.3%) | 3/33 (9.1%) |  | 0.229 | 0.1537 |
| Autoimmune disease | 0/30 (0%) | 1/33 (3.0%) | 1/14 (4.1%) | 3/33 (9.1%) |  | 0.207 | 0.2629 |

**Supplementary Data 5. GC-MS Metabolite abundance levels heatmaps in healthy, AM, moderate and severe COVID-19+ patients**. **Abbreviations:** 2-Aminobutanoic acid*: 2-Aminobutyric acid / alpha-aminobutyric acid. Butanoic acid*: Butanoic acid. 2-(methoxyimino)-3-methyl-. trimethylsilyl ester. Fumaric acid*: (E)-2-Butenedioic acid. 1,2-Dihydroxy-cyclohexene*: (R*,S*)- 3,8-Dioxa-2,9-disiladecane, 2,2,9,9-tetramethyl-5,6-bis[(trimethylsilyl)oxy]-. Oxoglutaric acid*: Alpha-ketoglutaric acid. 2-Hydroxyisovaleric acid*: 2-Hydroxy-3-methylbutyric acid / Pentanoic acid 2-[(trimethylsilyl)oxy]-, trimethylsilyl ester. Monostearin*: Monostearin / 1-stearoyl-rac-glycerol. Glycerol 3-phosphate*: Phosphoric acid. bis(trimethylsilyl) 2.3-bis[(trimethylsilyl)oxy]propyl ester. Octanoic acid*: (Z.Z)-9.12-Octadecadienoic acid. Vaccenic acid*: (E)-11-Octadecenoic acid. Elaidic acid*: (E)-Oleic acid. Oleic acid*: (Z)-Oleic Acid.

**
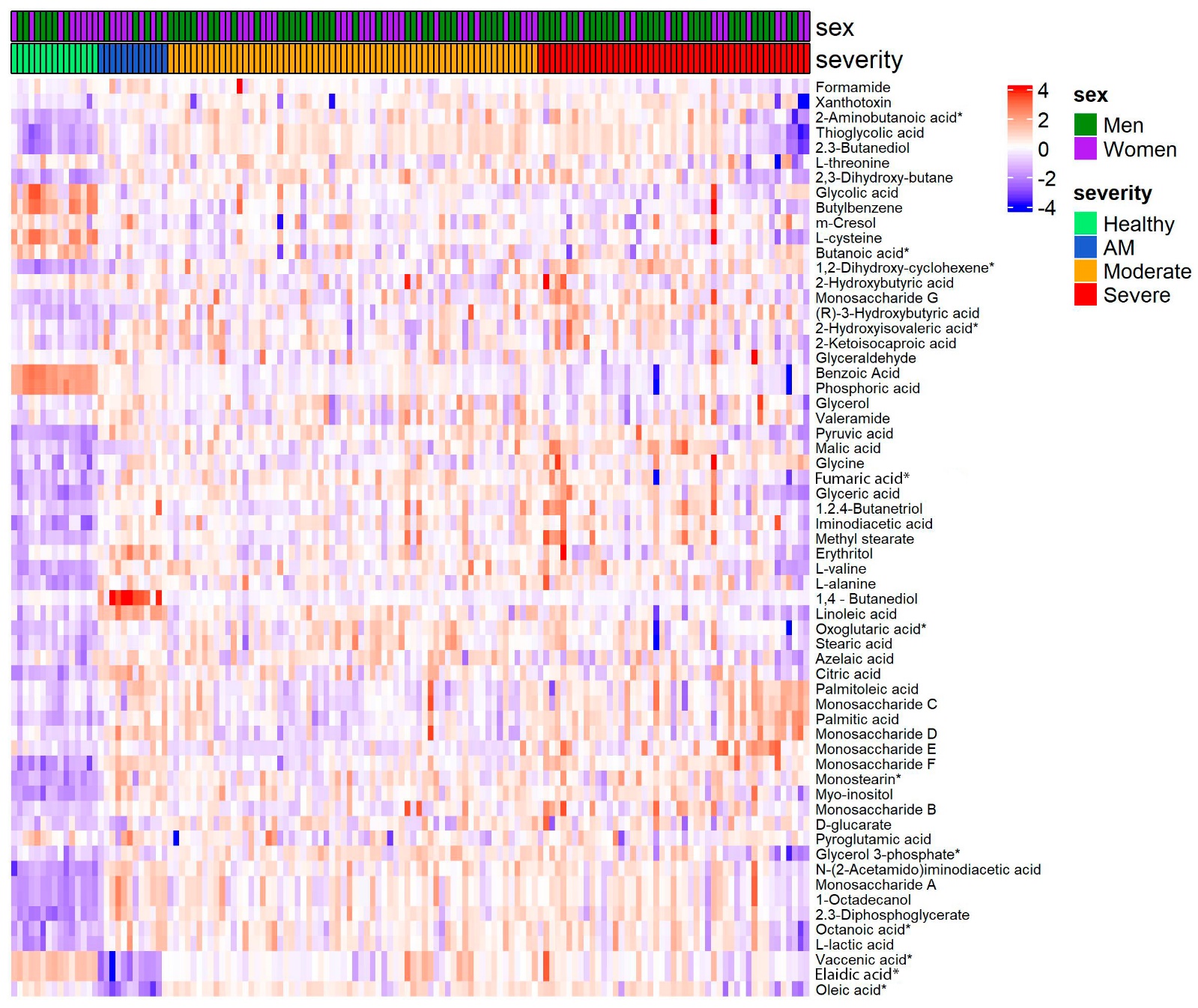
**

**Supplementary Data 6. CE-MS Metabolite concentration levels heatmaps in healthy, AM, moderate and severe COVID-19+ patients**. **Abbreviations:** Trans-3-hydroxyproline*: trans-3-hydroxyproline / trans-4-hydroxyproline / cis-4-Hydroxy-D-proline. L-leucine*: L-Leucine / Isoleucine. 1-Methyl-L-histidine*: 1-Methyl-L-histidine / 3-Methyl-L-histidine. D-alanyl-D-valine*: D-alanyl-D-valine / Glycyl-L-leucine / N2-acetyl-L-lysine. Isocitric acid*: Citric Acid / Isocitric acid

**
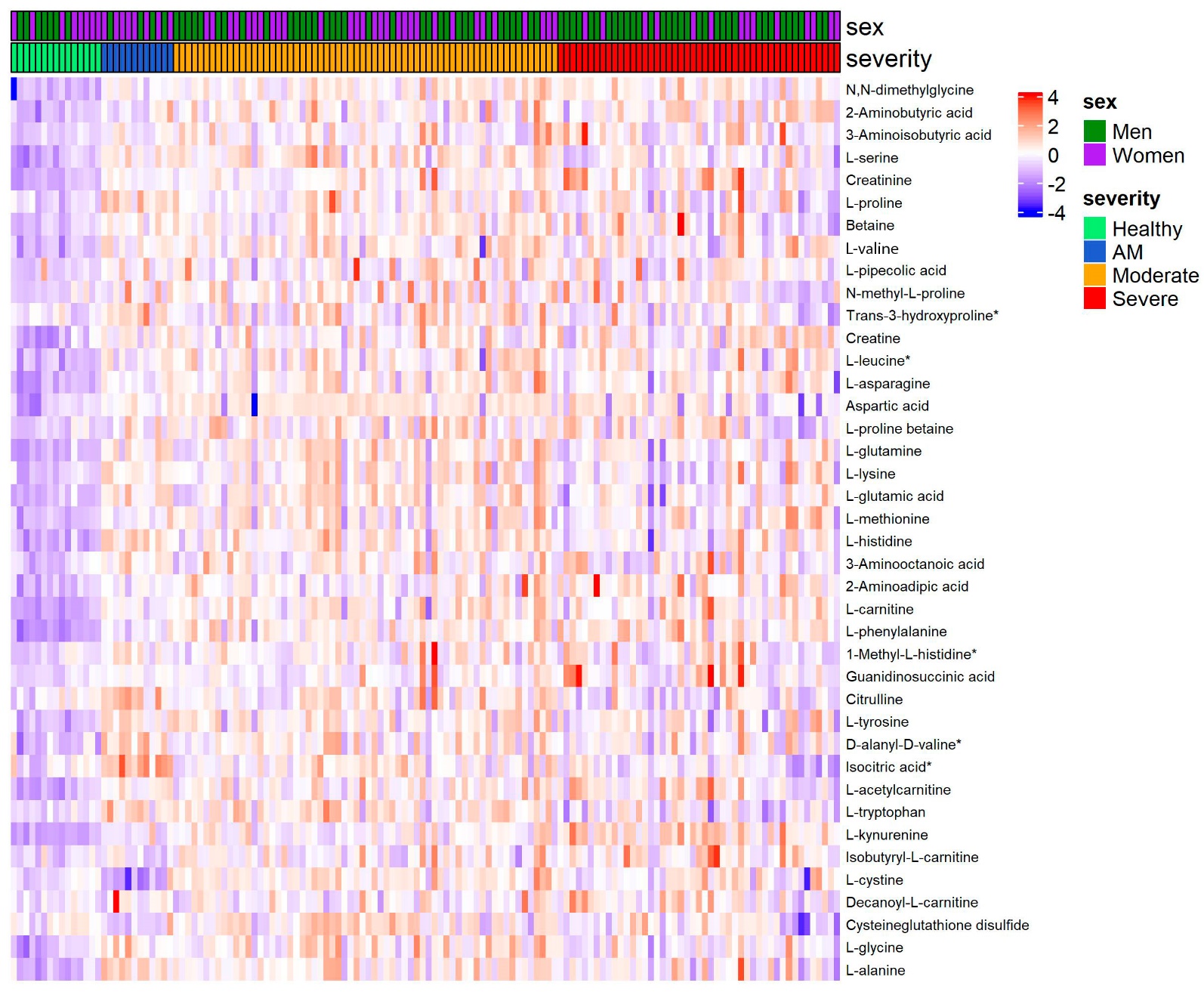
**

**Supplementary Data 7. Differential metabolites levels according to the different studied** **groups**. Volcano plots are shown for the log_2_ fold change, and the false discovery rate (FDR) obtained through non-parametric Kruskal-Wallis comparisons. Metabolites obtained by (A) GC-MS and (B) CE-MS are presented. Metabolites with a fold-change over 1.5 or below -1.5 with a *q*-value (FDR) value lower than 0.1 were considered as having significantly differential concentration. Metabolites with a log_2_ fold change below -05 are shown with blue dot. Metabolites with a log_2_ fold change above 0.5 are shown with a yellow dot. Metabolite name was added to those with a fold-change over 2 or below -2. **Abbreviations**: AM, Asymptomatic/Mild patients.


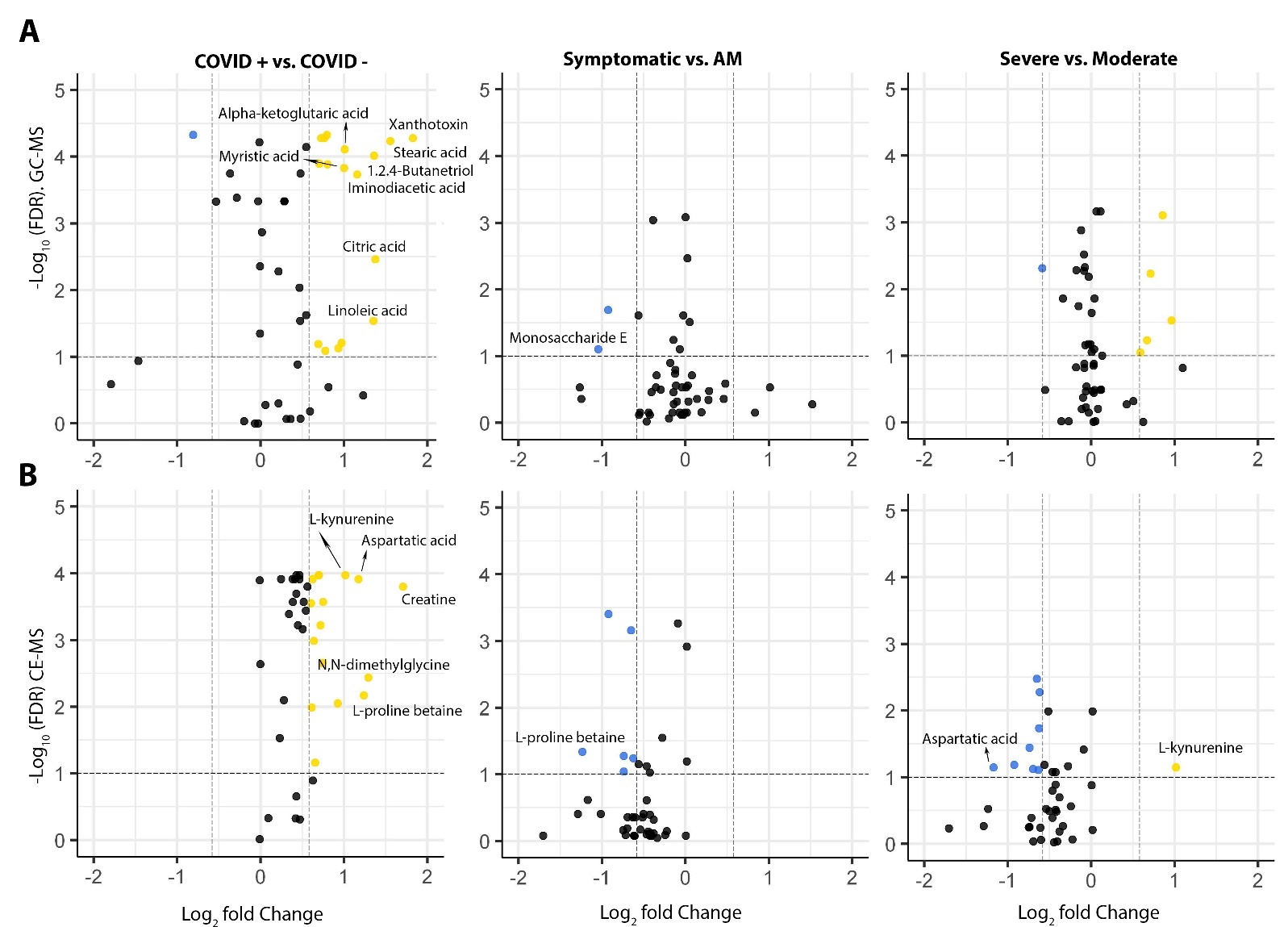


**Supplementary Data 8. Partial least squares - discriminant analysis (PLS-DA) scores plots of the different samples obtained through GC-MS and CE-MS**. (A) GC-MS and (B) CE-MS.

**
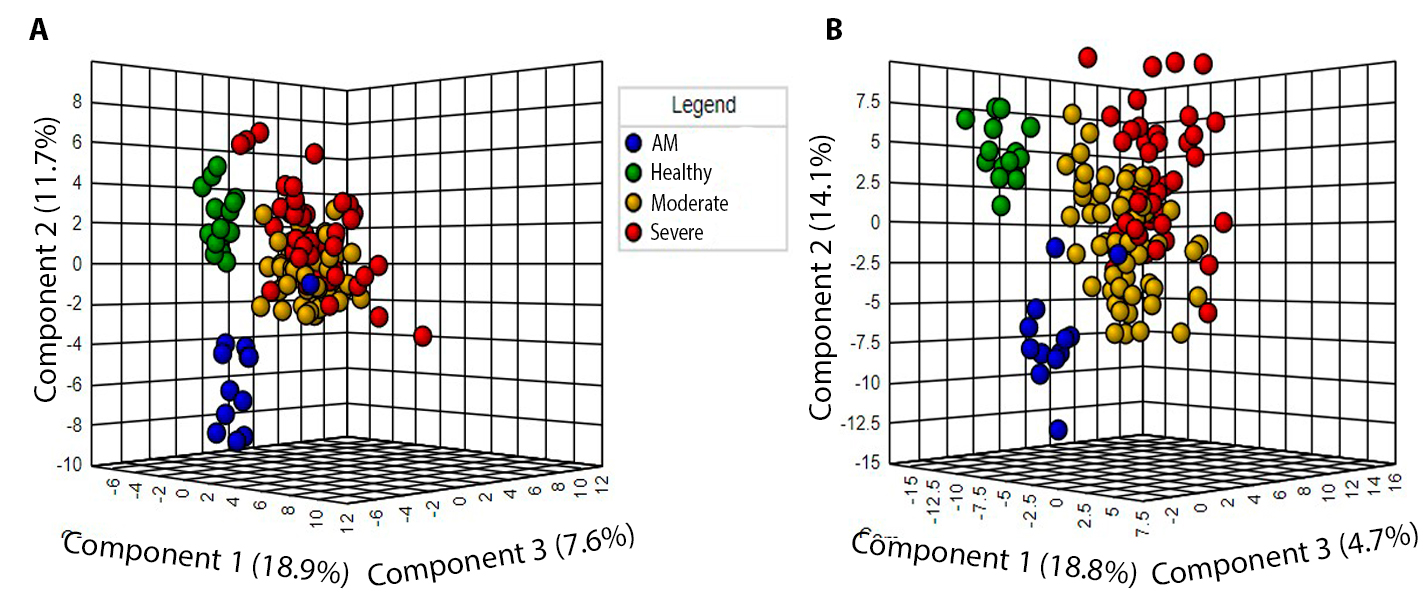
**

**Supplementary Data 9:** Results of cross-validation with leave-one-out (LOOCV) and permutation for each comparison.

| **GC-MS** | | | | |
| --- | --- | --- | --- | --- |
|  | **R^2^** | **Q^2^** | **Number of components** | **Prediction Accuracy During Training  method** |
| COVID-19 patients vs. Healthy controls | 0.939 | 0.882 | 5 | *p* < 0.001 |
| Asymptomatic/mild vs. Symptomatic | 0.775 | 0.665 | 3 | *p* < 0.001 |
| Severe vs. Moderate | 0.476 | 0.289 | 2 | *p* = 0.001 |
|  | | | | |
| **CE-MS** | | | | |
|  | **R^2^** | **Q^2^** | **Number of components** | **Prediction Accuracy During Training  method** |
| COVID-19 patients vs. Healthy controls | 0.925 | 0.827 | 5 | *p* < 0.001 |
| Asymptomatic/mild vs. Symptomatic | 0.869 | 0.703 | 4 | *p* < 0.001 |
| Severe vs. Moderate | 0.497 | 0.326 | 2 | *p* = 0.004 |

**Supplementary Data 10. Pathway analysis based on enrichment analysis procedure.** The False Discovery Rate (FDR) and the pathway impact are shown. Analysis was done with MetaboAnalyst software v 4.0.

| **Metabolic pathways** | **FDR** | **Impact** |
| --- | --- | --- |
| **COVID-19 patients vs. Healthy controls** |  |  |
| Phenylalanine, tyrosine and tryptophan biosynthesis | 7.07E-02 | 1.000 |
| Alanine, aspartate and glutamate metabolism | 1.73E-05 | 0.585 |
| D-glutamine and D-glutamate metabolism | 1.51E-02 | 0.500 |
| Glycine, serine and threonine metabolism | 5.72E-04 | 0.364 |
| Cysteine and methionine metabolism | 1.54E-02 | 0.264 |
| Citrate cycle (TCA cycle) | 5.77E-02 | 0.225 |
| Aminoacyl-tRNA biosynthesis (ARS) | 2.39E-10 | 0.167 |
| Glyoxylate and dicarboxylate metabolism | 1.51E-02 | 0.153 |
| Arginine biosynthesis | 2.36E-03 | 0.117 |
| Valine, leucine and isoleucine biosynthesis | 2.82E-02 | 0.000 |
| **Asymptomatic/mild vs. Symptomatic** |  |  |
| Phenylalanine tyrosine and tryptophan biosynthesis | 7.88E-02 | 0.502 |
| Cysteine and methionine metabolism | 7.88E-02 | 0.103 |
| Aminoacyl-tRNA biosynthesis (ARS) | 2.19E-04 | 0.023 |
| Alanine aspartate and glutamate metabolism | 7.88E-02 | 0.019 |
| **Moderate vs. Severe** |  |  |
| Alanine, aspartate and glutamate metabolism | 1.59E-04 | 0.579 |
| D-glutamine and D-glutamate metabolism | 2.14E-03 | 0.503 |
| Arginine biosynthesis | 9.97E-05 | 0.351 |
| Glycine, serine and threonine metabolism | 3.03E-02 | 0.241 |
| Histidine metabolism | 3.03E-02 | 0.218 |
| Aminoacyl-tRNA biosynthesis (ARS) | 2.23E-07 | 0.169 |
| Glyoxylate and dicarboxylate metabolism | 3.20E-03 | 0.154 |
| Nitrogen metabolism | 4.74E-02 | 0.012 |

**Supplementary Data 11. Venn diagram of dysregulated metabolites across pairwise comparisons for GC-MS and CE-MS** by multivariable logistic regression models. Significant metabolites, identified by multivariable logistic regression and shared by different groups are listed. **Abbreviations**: Oxoglutaric acid*: Alpha-ketoglutaric acid. 2-Hydroxyisovaleric acid*: 2-Hydroxy-3-methylbutyric acid / Pentanoic acid 2-[(trimethylsilyl)oxy]-, trimethylsilyl ester. Monostearin*: Monostearin / 1-stearoyl-rac-glycerol. Isocitric acid*: Citric Acid / Isocitric acid. Trans-3-hydroxyproline*: trans-3-hydroxyproline / trans-4-hydroxyproline / cis-4-Hydroxy-D-proline.

**
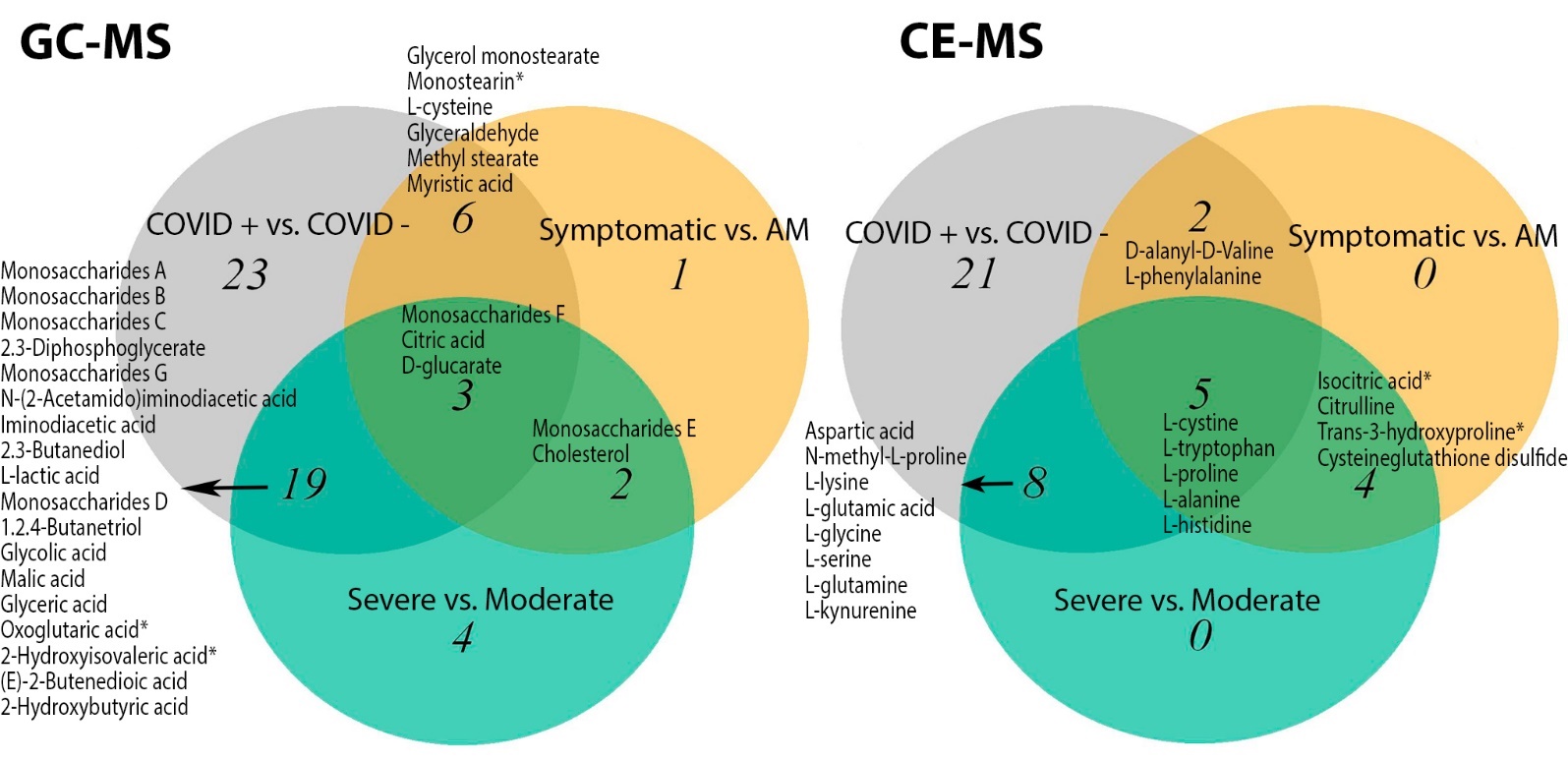
**

**Supplementary Data 12. Correlations between metabolite and inflammatory biomarkers in COVID-19 patients**. Chord diagrams depicting significant correlations between significant dysregulated metabolites and cytokines. Chords are color-coded: blue, negatively correlated, and red, positively correlated. All significant correlations (*q*-value <0.1) were included. **Abbreviations**: Trans-3-hydroxyproline*: trans-3-hydroxyproline / trans-4-hydroxyproline / cis-4-Hydroxy-D-proline. Monostearin*: Monostearin / 1-stearoyl-rac-glycerol. D-alanyl-D-valine*: D-Alanyl-D-Valine / Glycyl-L-leucine / N2-Acetyl-L-Lysine: 2-Hydroxyisovaleric acid*: 2-Hydroxy-3-methylbutyric acid / Pentanoic acid 2-[(trimethylsilyl)oxy]-, trimethylsilyl ester. 1-Methyl-L-histidine*: 1-Methyl-L-histidine / 3-Methyl-L-histidine. 1,4 - Butanediol*: (R*,S*)- 3,8-Dioxa-2,9-disiladecane, 2,2,9,9-tetramethyl-5,6-bis[(trimethylsilyl)oxy]. Elaidic acid*: (E)-Oleic acid.


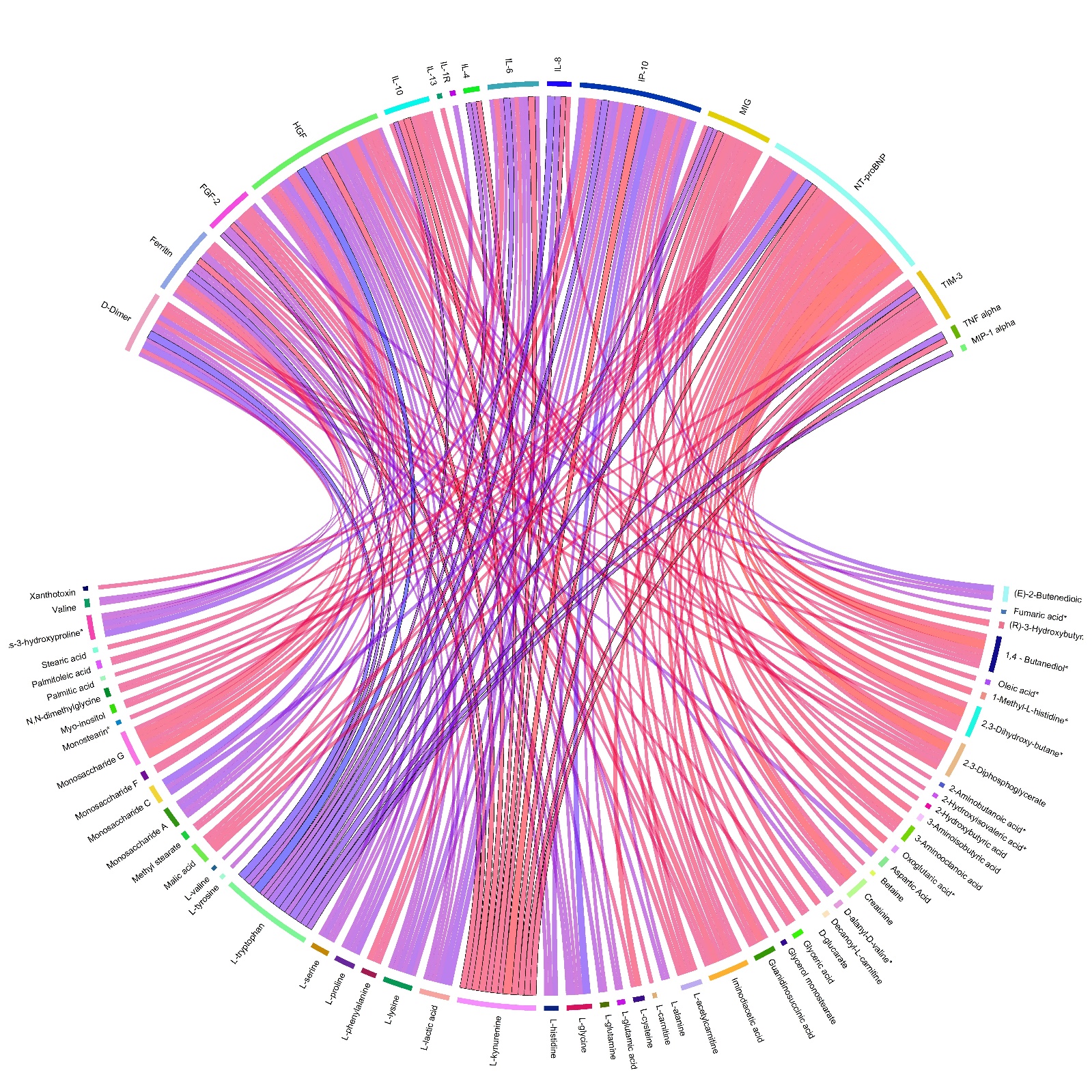


**Supplementary Data 13. Correlations between metabolite and inflammatory biomarkers in symptomatic COVID-19 individuals**. Chord diagrams depicting significant correlations between significant dysregulated metabolites and cytokines. Chords are color-coded: blue, negatively correlated, and red, positively correlated. All significant correlations (*q*-value <0.1) were included. **Abbreviations**: Monostearin*: Monostearin / 1-stearoyl-rac-glycerol.


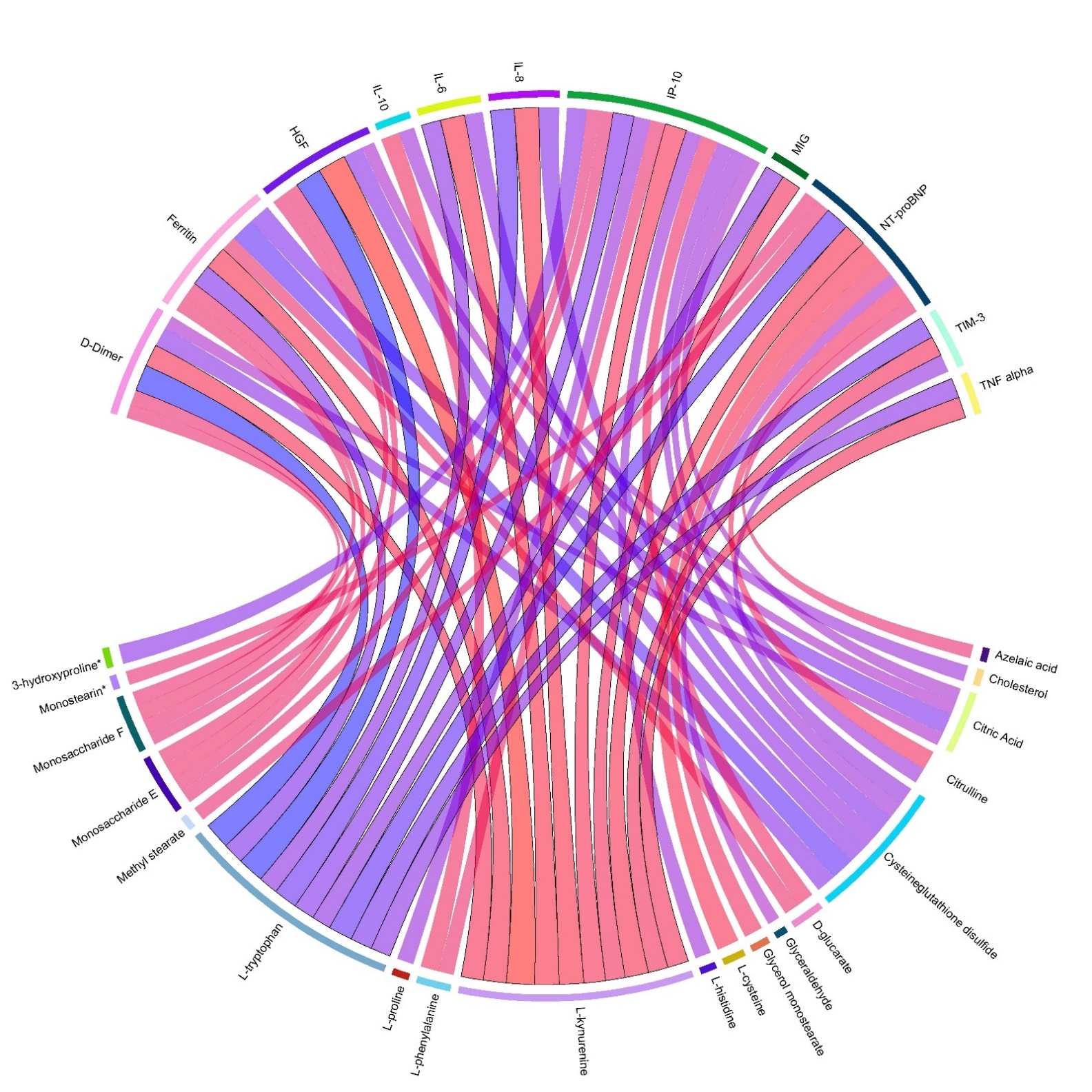


**Supplementary Data 14. Correlations between metabolite and inflammatory markers in moderate COVID-19 individuals**. Chord diagrams depicting significant correlations between significant dysregulated metabolites and cytokines. Chords are color-coded: blue, negatively correlated, and red, positively correlated. All significant correlations (*q-*value <0.1) were included. **Abbreviations**: Oxoglutaric acid*: Alpha-ketoglutaric acid


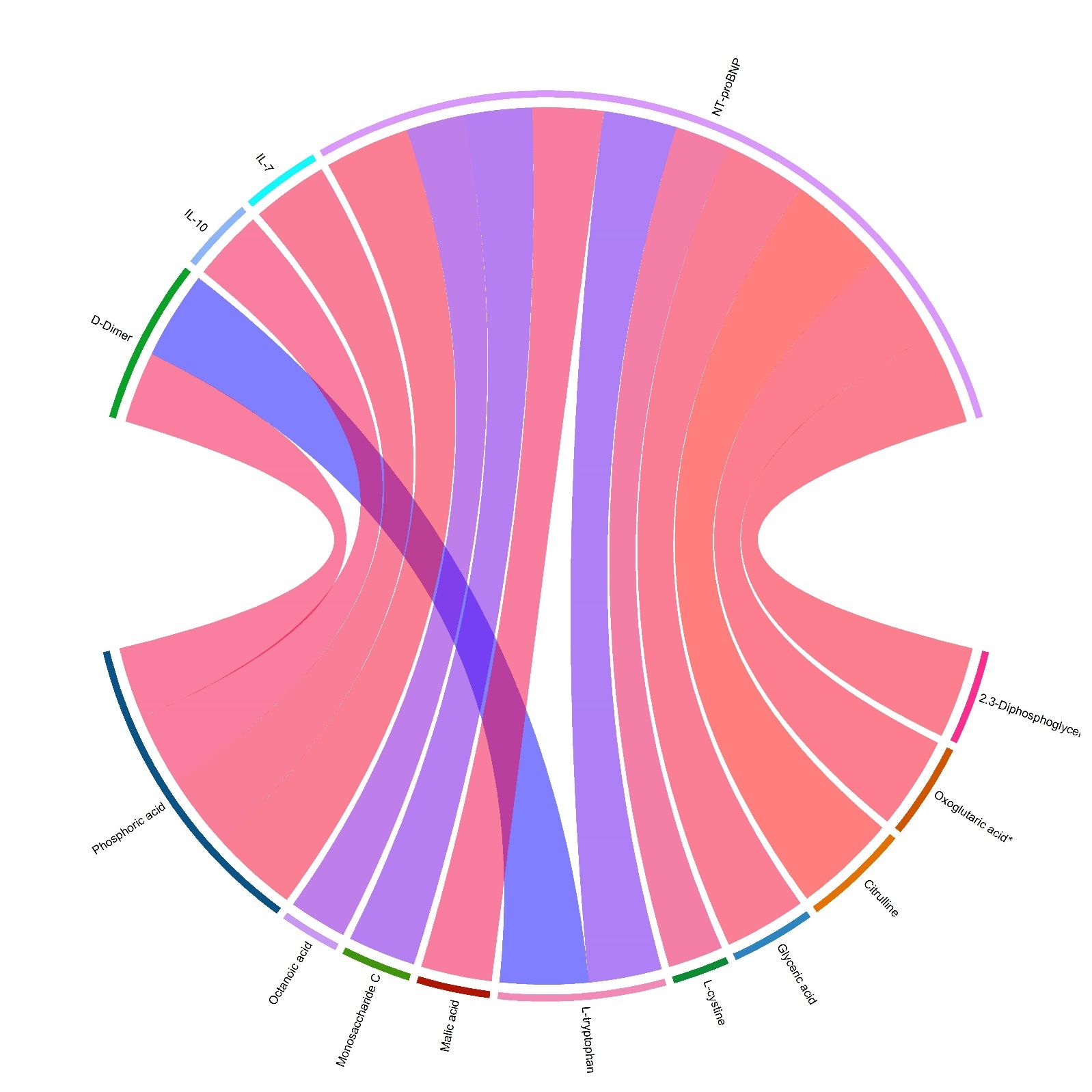


**Supplementary Data 15. Sex interaction for metabolites.** Interaction was tested using multiple logistic models by adding “sex:metabolite” along with each metabolite, age and sex. In this table, log OR, standard error, and FDR are shown for the interaction of each metabolite with sex. **Abbreviations**: OR: odd ratio. FDR: false discovery rate. SE: standard error**.** 1,4 - Butanediol*: (R*,S*)- 3,8-Dioxa-2,9-disiladecane, 2,2,9,9-tetramethyl-5,6-bis[(trimethylsilyl)oxy]. Glycerol 3-phosphate*: Phosphoric acid. bis(trimethylsilyl) 2.3-bis[(trimethylsilyl)oxy]propyl ester. 2-Hydroxyisovaleric acid*: 2-Hydroxy-3-methylbutyric acid / Pentanoic acid 2-[(trimethylsilyl)oxy]-, trimethylsilyl ester. Butanoic acid*: Butanoic acid. 2-(methoxyimino)-3-methyl-. trimethylsilyl ester. Carbamate*: Tris(trimethylsilyl)carbamate. Trans-3-hydroxyproline*: trans-3-hydroxyproline / trans-4-hydroxyproline / cis-4-Hydroxy-D-proline.

|  | **Log OR** | **SE** | **FDR** |
| --- | --- | --- | --- |
| **GC-MS** |  |  |  |
| 1,4 - Butanediol* | 0.981 | 0.390 | 0.053 |
| 1.2.4-Butanetriol | 1.574 | 0.449 | 0.007 |
| 1-Octadecanol | 0.746 | 0.358 | 0.090 |
| Glycerol 3-phosphate* | 0.670 | 0.315 | 0.089 |
| 2.3-Butanediol | -1.001 | 0.405 | 0.053 |
| 2-Hydroxyisovaleric acid* | 0.639 | 0.269 | 0.060 |
| 2-Hydroxybutyric acid | 0.878 | 0.316 | 0.036 |
| Benzoic Acid | 0.580 | 0.270 | 0.087 |
| Butanoic acid* | -0.890 | 0.423 | 0.090 |
| Butylbenzene | -1.320 | 0.448 | 0.024 |
| D-glucarate | 2.263 | 0.586 | 0.004 |
| Erythritol | 0.901 | 0.359 | 0.053 |
| Formamide | -1.370 | 0.622 | 0.080 |
| Glyceric acid | 1.190 | 0.341 | 0.007 |
| L-lactic acid | -1.037 | 0.401 | 0.052 |
| Monosaccharide B | 1.090 | 0.333 | 0.012 |
| Monosaccharide C | 1.180 | 0.339 | 0.007 |
| Monosaccharide D | 1.808 | 0.430 | 0.002 |
| Monosaccharide E | 0.679 | 0.297 | 0.070 |
| Monosaccharide F | 0.942 | 0.312 | 0.021 |
| Monosaccharide G | 1.212 | 0.380 | 0.013 |
| Myo-inositol | 0.834 | 0.325 | 0.052 |
| Octanoic acid | 0.875 | 0.393 | 0.077 |
| Phosphoric acid | -1.539 | 0.590 | 0.052 |
| Thioglycolic acid | -1.152 | 0.485 | 0.060 |
| Carbamate* | -1.501 | 0.606 | 0.053 |
| Valeramide | -1.054 | 0.446 | 0.060 |
| **CE-MS** |  |  |  |
| Aspartic acid | -0.752 | 0.309 | 0.074 |
| Citrulline | -0.772 | 0.300 | 0.074 |
| Cysteineglutathione disulfide | -0.743 | 0.284 | 0.074 |
| L-glycine | -0.709 | 0.299 | 0.079 |
| L-kynurenine | 1.222 | 0.361 | 0.029 |
| L-serine | -0.758 | 0.300 | 0.074 |
| L-tryptophan | -0.973 | 0.311 | 0.035 |
| Trans-3-hydroxyproline* | -0.775 | 0.299 | 0.074 |

**Supplementary Data 16. Sex interaction for plasma biomarkers.** The interaction was tested in multiple logistic models by adding “sex:plasma-marker” along with each marker, age, and sex. In this table, log OR, standard error, and FDR are shown for the interaction of each metabolite with sex. **Abbreviations**: OR: odd ratio. FDR: false discovery rate. SE: standard error**.**

|  | **Log OR** | **SE** | **FDR** |
| --- | --- | --- | --- |
| FGF-2 | 10.996 | 4.562 | 0.032 |
| G-CSF | 10.540 | 4.889 | 0.045 |
| HGF | 8.997 | 2.697 | 0.004 |
| IFN gamma | 4.654 | 2.216 | 0.048 |
| IL-10 | 12.085 | 4.141 | 0.010 |
| IL-12 | 12.573 | 6.025 | 0.048 |
| IL-1RA | 15.997 | 7.733 | 0.048 |
| IL-2 | 5.380 | 3.143 | 0.094 |
| IL-4 | 5.033 | 1.952 | 0.024 |
| IL-6 | 5.899 | 1.723 | 0.004 |
| IL-7 | 7.589 | 3.291 | 0.037 |
| IL-8 | 10.139 | 2.697 | 0.004 |
| M-CSF | 4.708 | 2.167 | 0.045 |
| MCP-1 | 3.850 | 1.673 | 0.037 |
| MIG | 4.663 | 1.420 | 0.004 |
| MIP-1 | 8.391 | 2.557 | 0.004 |
| NT-proBNP | 3.834 | 1.300 | 0.010 |
| TIM-3 | 5.986 | 2.243 | 0.020 |
| TNF alpha | 15.780 | 4.530 | 0.004 |


Supplement: Supplementary file 1 [file DataSheet_1.docx]
